# Inferring Epistasis from Genetic Time-series Data

**DOI:** 10.1093/molbev/msac199

**Published:** 2022-09-21

**Authors:** Muhammad Saqib Sohail, Raymond H Y Louie, Zhenchen Hong, John P Barton, Matthew R McKay

**Affiliations:** Department of Electronic and Computer Engineering, Hong Kong University of Science and Technology, Hong Kong SAR, People’s Republic of China; The Kirby Institute, University of New South Wales, Sydney, New South Wales, Australia; Department of Physics and Astronomy, University of California, Riverside, CA, USA; Department of Physics and Astronomy, University of California, Riverside, CA, USA; Department of Computational and Systems Biology, University of Pittsburgh School of Medicine, Pittsburgh, PA, USA; Department of Electronic and Computer Engineering, Hong Kong University of Science and Technology, Hong Kong SAR, People’s Republic of China; Department of Chemical and Biological Engineering, Hong Kong University of Science and Technology, Hong Kong SAR, People’s Republic of China; Department of Electrical and Electronic Engineering, University of Melbourne, Melbourne, Victoria, Australia; Department of Microbiology and Immunology, University of Melbourne, at The Peter Doherty Institute for Infection and Immunity, Melbourne, Victoria, Australia

**Keywords:** Bayesian inference, selection, epistasis, linkage, path integral, diffusion, time-series data, longitudinal data

## Abstract

Epistasis refers to fitness or functional effects of mutations that depend on the sequence background in which these mutations arise. Epistasis is prevalent in nature, including populations of viruses, bacteria, and cancers, and can contribute to the evolution of drug resistance and immune escape. However, it is difficult to directly estimate epistatic effects from sampled observations of a population. At present, there are very few methods that can disentangle the effects of selection (including epistasis), mutation, recombination, genetic drift, and genetic linkage in evolving populations. Here we develop a method to infer epistasis, along with the fitness effects of individual mutations, from observed evolutionary histories. Simulations show that we can accurately infer pairwise epistatic interactions provided that there is sufficient genetic diversity in the data. Our method also allows us to identify which fitness parameters can be reliably inferred from a particular data set and which ones are unidentifiable. Our approach therefore allows for the inference of more complex models of selection from time-series genetic data, while also quantifying uncertainty in the inferred parameters.

Epistasis refers to fitness effect of mutant alleles that differ from the sum of the fitness effects of each individual mutant (Carlborg and Haley 2004; Phillips 2008; de Visser et al. 2011; Lehner 2011). Epistasis therefore causes the fitness effect of a mutation to depend on the genetic background on which it arises. Theoretical and experimental studies have shown that epistasis can play a role in speciation (Wade 2002; Gavrilets 2004) and adaptation (Chou et al. 2011; Hansen 2013), and that it is intertwined with the evolutionary advantages of recombination (de Visser and Elena 2007; Kouyos et al. 2007). Epistasis is not uncommon in nature, and signatures of strong epistasis have been observed in lab evolution and site-directed mutagenesis experiments (Bershtein et al. 2006; Khan et al. 2011; Salverda et al. 2011; Gong et al. 2013).

Epistasis makes fitness landscapes more complex, shaping evolution (Phillips 2008; de Visser and Krug 2014). For example, epistasis may make certain mutational pathways more difficult to traverse while others become more readily accessible, depending on the sequence background (Weinreich et al. 2005, 2006; Phillips 2008; Salverda et al. 2011; de Visser and Krug 2014; Pedruzzi et al. 2018). A better understanding of epistasis could therefore help to characterize the evolutionary dynamics of novel viral strains capable of evading immune responses (Illingworth et al. 2014), pathogens that develop drug resistance (Hughes and Andersson 2015; Zhang et al. 2020) and tumor growth in cancers (Yates and Campbell 2012; Wang et al. 2014), as well as the adaptation of populations under lab settings (Domínguez-García et al. 2019).

Advances in sequencing technologies over the past decades have made it possible to obtain detailed, time-resolved population-level sequence data, enabling the study of evolving populations in fine detail. Examples of such data include those obtained from evolving populations in vitro (Barrick et al. 2009), ones sampled from naturally-infected hosts (Murcia et al. 2012; Illingworth 2015; Zanini et al. 2015; Xue et al. 2017), and time-resolved global influenza evolutionary records (Bao et al. 2008). These evolving populations contain multiple polymorphic loci, making the epistasis between mutant alleles a potential factor in their evolution.

A complicating factor in inferring epistasis from such time-series data is the presence of linkage effects. Genetic linkage can arise by chance as a consequence of shared inheritance, or for functional reasons due to epistatic interactions between linked loci. Linkage can be especially strong when recombination is low, selection is strong, and novel mutations frequently appear and compete in a population (Desai and Fisher 2007; Neher and Shraiman 2009; Sniegowski and Gerrish 2010). The ability to distinguish the effects of epistasis from linkage due to chance is therefore important for the reliable inference of fitness from genetic time-series data.

The large majority of existing methods for inferring the fitness effects of mutations from genetic data ignore epistasis in their modeling. Hence they do not estimate epistasis, nor do they account for epistatic effects when estimating the fitness advantage of an allele. Most existing methods are based on single-locus models which assume independent evolution of loci (Bollback et al. 2008; Malaspinas et al. 2012; Mathieson and McVean 2013; Feder et al. 2014; Lacerda and Seoighe 2014; Steinrücken et al. 2014; Foll et al. 2015; Topa et al. 2015; Ferrer-Admetlla et al. 2016; Gompert 2016; Schraiber et al. 2016; Iranmehr et al. 2017; Taus et al. 2017; Zinger et al. 2019), thus they are unable to directly account for genetic linkage or epistasis. A few methods (Illingworth and Mustonen 2011; Terhorst et al. 2015; Sohail et al. 2021) have been developed that consider the joint evolution of multiple loci, but these assume additive fitness models. Hence, while they account for genetic linkage, they do not consider epistasis. A notable exception are the methods that use an extension of the multi-locus approach of Illingworth and Mustonen (2011) to account for epistatic interactions (Illingworth et al. 2014; Illingworth 2015). These fit a deterministic evolutionary model based on observed genotype frequencies, and while presenting an important advance, they require the use of computationally intensive numerical optimization methods.

## New Approaches

Here we present a novel method that provides a closed-form, analytical solution for estimates of selection coefficients and pairwise epistatic interactions from genetic time-series data. Due to its analytical form, our approach is straightforward to implement and computationally efficient for moderate numbers of loci. Our method is based on an extension of the marginal path likelihood (MPL) framework (Sohail et al. 2021) to account for epistasis. We use a path integral method derived from statistical physics (Risken 1989) to efficiently represent the likelihood of an observed trajectory of single and double mutant allele frequencies. We then apply Bayesian theory to estimate the fitness parameters that best explain an observed evolutionary trajectory.

We model a population evolving under the Wright–Fisher (WF) model with mutation, selection, and recombination. First, we define x(t) as the vector of single and double mutant allele frequencies observed at generation *t*. For a system with *L* loci labeled by i=1,2,…,L, the first *L* entries of x(t) represent mutant allele frequencies $x_{i}(t)$, and entries from L+1 to R=L(L+1)/2 represent the frequencies of individuals in the populations with mutant alleles at loci *i* and *j*, denoted $x_{ij}(t)$. Under WF dynamics the probability of observing a trajectory or ‘path’ $\left(x(t_{1}),x(t_{2}),\ldots ,x(t_{K})\right)$ conditioned on $x(t_{0})$ is given by

(1) $$P((x(t_{k}){)}_{k=1}^{K}\;|\;x(t_{0}))=\prod_{k=0}^{K-1}P(x(t_{k+1})\;|\;x(t_{k})).$$

We approximate the probability in (1) with a path integral. The first step of this approach is to approximate the WF process by a diffusion process (Kimura 1964; Ewens 2012; Tataru et al. 2015; He et al. 2017; Tataru et al. 2017). Under this approximation, the transition probabilities that appear on the right-hand side of (1) can be approximated by the transition probability density, ϕ, of a diffusion process (Durrett 2008), multiplied by a constant scaling term. In principle, $P(x(t_{k+1})\;|\;x(t_{k}))$ can be approximated using numerical integration techniques to solve the diffusion equations (Bollback et al. 2008; Malaspinas et al. 2012; Ferrer-Admetlla et al. 2016). Such approaches, however, are computationally intensive and lead to expressions that are difficult to treat analytically, even at the single locus level. Instead, the path integral approach we take allows for efficient computation of (1) by discretizing the transition probability density for small time steps.

Taking a Gaussian prior for the selection coefficients and epistasis parameters and applying the maximum a posteriori criterion, we obtain an analytical expression for the estimates of selection coefficients and epistasis terms (collected into a vector $\hat{s}$) given the observed allele frequency trajectories (see Materials and Methods for details):

(2) $$\hat{s}=[C_{\mathrm{int}}+\gamma I{]}^{-1}\times [\Delta x-\mu \;v_{\mathrm{int}}-r\;\eta_{\mathrm{int}}].$$

Here $C_{\mathrm{int}}$ is the covariance matrix of single and double mutant allele frequencies integrated over time, γ is a regularization parameter, and *I* is the identity matrix. The net change in single and double mutant allele frequencies over the trajectory is denoted by Δx. Entries of the $v_{\mathrm{int}}$ and $\eta_{\mathrm{int}}$ vectors, which describe the flux in mutant allele frequencies due to mutation and recombination, respectively, are given by

(3) $$v_{e}(x(t_{k}))=\left\{ \begin{array}{ll} 1-2x_{i}(t_{k}) & 1\leq e\leq L\\ x_{i}(t_{k})+x_{\;j}(t_{k})-4x_{ij}(t_{k}) & L<e\leq R, \end{array}\right.$$

and

(4) $$\eta_{e}(x(t_{k}))=\left\{ \begin{array}{ll} 0 & 1\leq e\leq L\\ \left(i-j)(x_{ij}(t_{k})-x_{i}(t_{k})x_{\;j}(t_{k})\right) & L<e\leq R, \end{array}\right.$$

integrated over time. Here e↦i for e≤L and e↦(i,j) for L<e≤R. We use μ for the mutation probability per locus per generation (assuming for simplicity that the probability to mutate from mutant to wild-type (WT) is the same as that to mutate from WT to mutant, though we relax this assumption in supplementary text, Supplementary Material online), and *r* is the recombination probability per locus per generation.

Intuitively, (2) shows that excess changes in allele frequencies/correlations that cannot be explained by mutation or recombination are evidence for selection/epistasis. The first term in the ‘numerator’, Δx, gives the observed change in the single and double mutant allele frequencies between the final and the initial time points. This raw difference is then adjusted by the expected cumulative mutational flows of single and double mutant frequencies over the trajectory, $\mu \;v_{\mathrm{int}}$. Finally, the change in double mutant frequencies is adjusted to account for their expected shift due to recombination, $r\;\eta_{\mathrm{int}}$. Linkage effects are incorporated through the inverse of the regularized integrated covariance matrix. This matrix, which captures the magnitude of allele frequency changes expected due to chance alone, also sets the scale of the inferred selection coefficients and epistatic interactions. Frequency changes that are significantly larger than chance expectations are therefore evidence for strong selection. As an example, distant loci that remain strongly linked over long times despite frequent recombination (see (4)) would suggest a strong, positive epistatic interaction between these loci.

In the following, we use simulations to demonstrate that our approach accurately infers fitness parameters using data from populations evolving under selection, mutation, recombination, epistasis, and nontrivial genetic linkage. We also show conditions under which reliable inference of selection and epistasis is possible. In cases where low genetic diversity precludes the accurate inference of some fitness parameters, MPL is still able to infer their collective fitness contributions.

## Results

### Accurate Estimation of Epistasis and Selection Coefficients

We first analyzed the performance of MPL on a two-locus bi-allelic system. We ran extensive simulations varying the selection strength, the composition of the initial population and different types of epistasis. The types of epistatic interactions we considered include positive epistasis, where the double mutant has a fitness higher than the sum of the individual fitness effects of each mutant allele; negative epistasis, where the fitness of the double mutant is lower than the sum of the individual fitness effects of each mutant allele; and sign epistasis, where the direction and the magnitude of the fitness effect of epistasis is opposite to and larger than the sum of the individual fitness effect of the two mutant alleles.

**Fig. 1.**
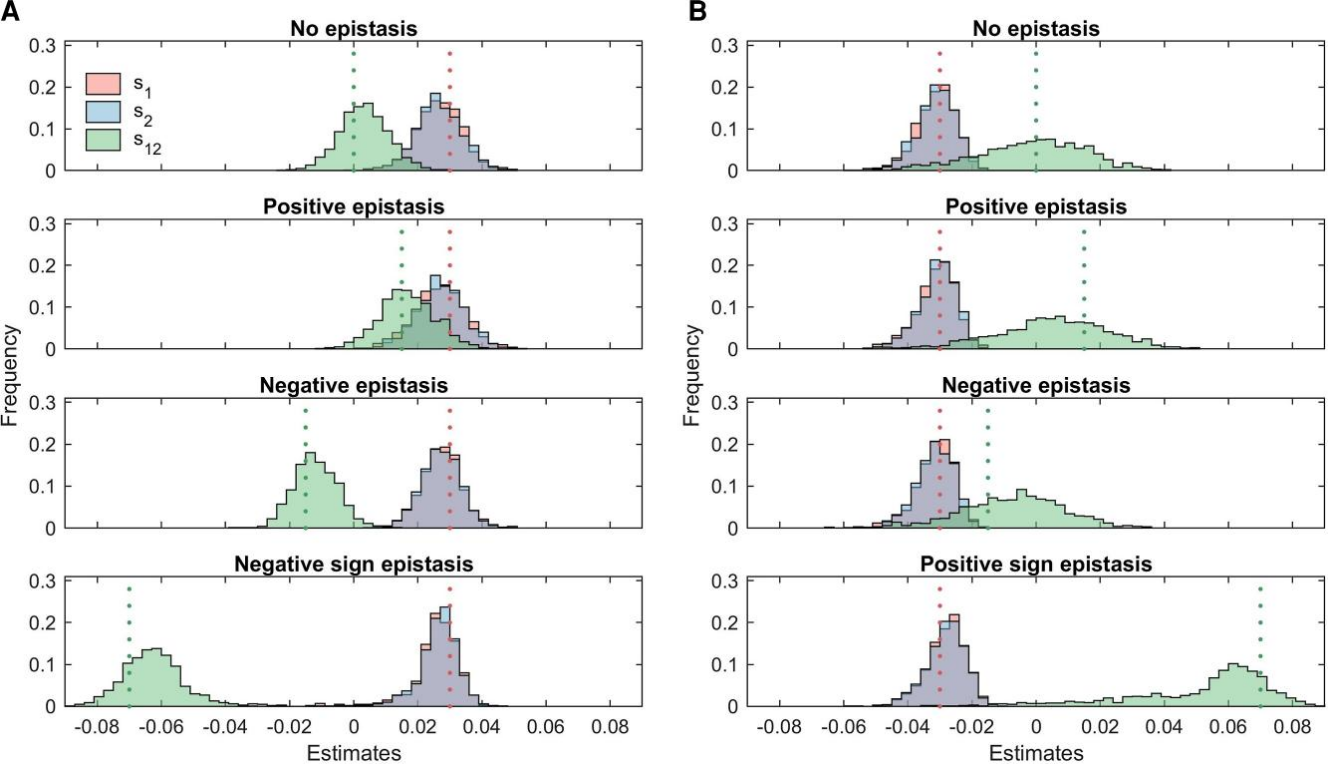
MPL can accurately infer selection coefficients and pairwise epistasis terms. Results were obtained for a two-locus system with selection, mutation, and recombination. (*A*) shows distribution of inferred selection coefficients and pairwise epistasis terms for various forms of epistasis when both selection coefficients are positive ($s_{1}=s_{2}=0.03$), while (*B*) shows the same for the case when both selection coefficients are negative ($s_{1}=s_{2}=-0.03$). The pairwise epistasis term $s_{12}$ was set to {0,0.015,−0.015,0.07,−0.07} to simulate the scenarios of no epistasis, positive epistasis, negative epistasis, positive sign epistasis, and negative sign epistasis, respectively. Other simulation parameters included per locus mutation probability $\mu =10^{-3}$, per locus recombination probability $r=10^{-3}$, and population size N=1000. The initial population consisted of only the WT genotype (00). The sampling parameters were set to $n_{s}=100$, Δt=10, and T=1000, where $n_{s}$ is the number of samples, Δt is the time sampling step and *T* is the number of generations used for inference. All simulation results were computed over 1000 Monte Carlo runs. The dashed lines represent the true selection coefficients ($s_{1}$ and $s_{2}$) and epistasis term ($s_{12}$). In these simulations, $s_{1}=s_{2}$, hence the histograms of the estimates of the two have a significant overlap shown in grey color.

**Fig. 2.**
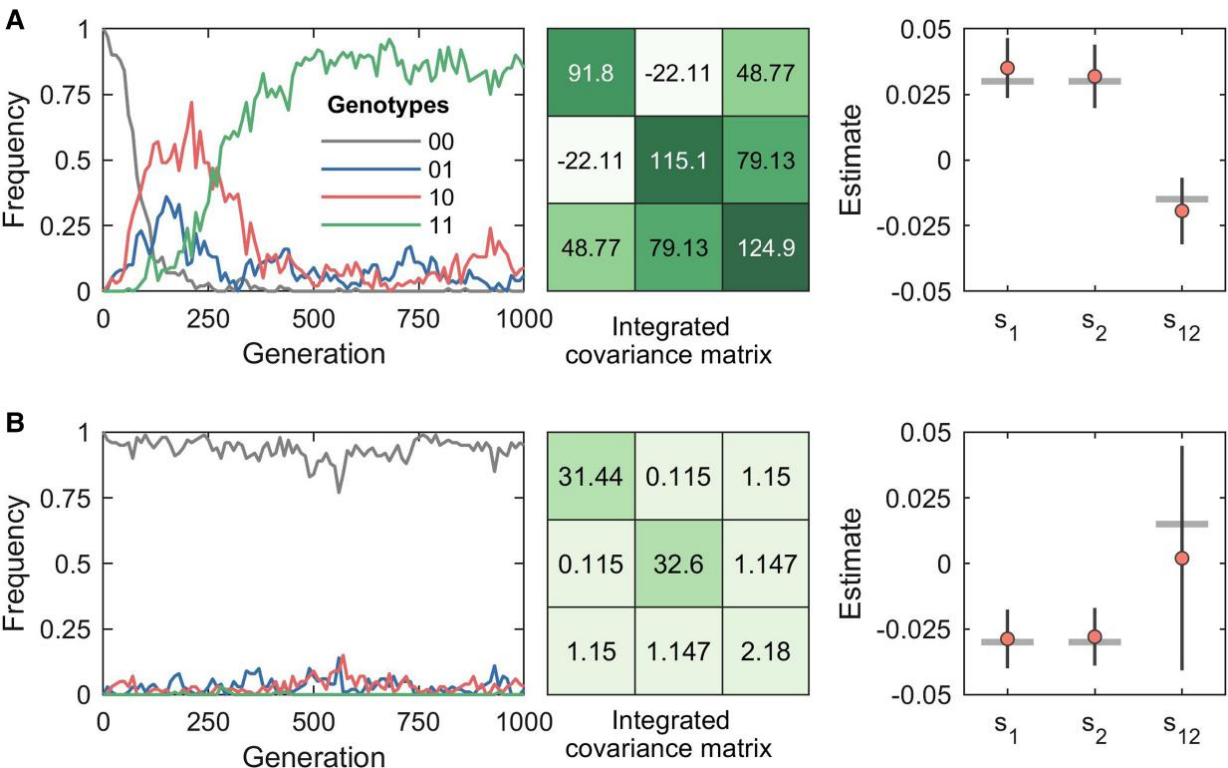
Higher genetic diversity leads to more accurate inference. (*A*) shows a sample run (*left* panel) of a two-locus system (negative epistasis scenario) where all genotypes are well represented in the data, as indicated by the magnitude of the diagonal entries of the integrated covariance matrix (*center* panel). This leads to accurate estimation of the epistasis term and the selection coefficients (*right* panel). The vertical bars in the *right* panel indicate the 95% confidence intervals while the horizontal bars indicate the true selection coefficients and epistasis terms. (*B*) shows a sample run (*left* panel) of a two-locus system (positive epistasis scenario) where the double mutant genotype has limited diversity, as indicated by the magnitude of the bottom right entry of the integrated covariance matrix (*center* panel). This leads to low accuracy in the estimate of the epistasis term. The selection coefficient estimates are still accurate because the single mutant genotypes, although present at low frequencies, are well represented in the data as indicated by the first two entries of the diagonal of the integrated covariance matrix (*center* panel). The results were obtained for a two-locus system with selection, mutation, and recombination. We set the selection coefficients $s_{1}$, $s_{2}$ and epistasis term $s_{12}$ to {0.03,0.03,−0.015} and {−0.03,−0.03,0.015} in (*A*) and (*B*), respectively. Other system parameters included per locus mutation probability $\mu =10^{-3}$, per locus recombination probability $r=10^{-3}$, and population size N=1000. The initial population consisted of only the WT genotype. The sampling parameters, in both simulations, were set to $n_{s}=100$, Δt=10, and T=1000, where $n_{s}$ is the number of samples, Δt is the time sampling step and *T* is the number of generations used for inference.

We found that MPL is typically able to accurately infer underlying fitness parameters. In the simulation shown in figure 1, the initial population consisted of only the WT genotype. MPL estimates (21) of selection coefficients were accurate in each simulated scenario. Estimates of the epistasis terms were better in scenarios where both the selection coefficients were beneficial (fig. 1*A*) compared with the scenarios where both were deleterious (fig. 1*B*), regardless of the type of epistasis. This is because double mutants tend to appear very rarely in cases where both single mutants were less fit than WT, as the single mutants are rapidly purged from the population. In such cases (fig. 1*B*), the double mutant genotype never exceeded 4% of the population in our simulations. A similar situation occurred in the positive sign epistasis scenario (fig. 1*Bbottom* panel). Thus, genetic diversity constrains the accuracy of the epistasis estimates, which is also reflected in the uncertainty of the inferred parameters (fig. 2).

**Fig. 3.**
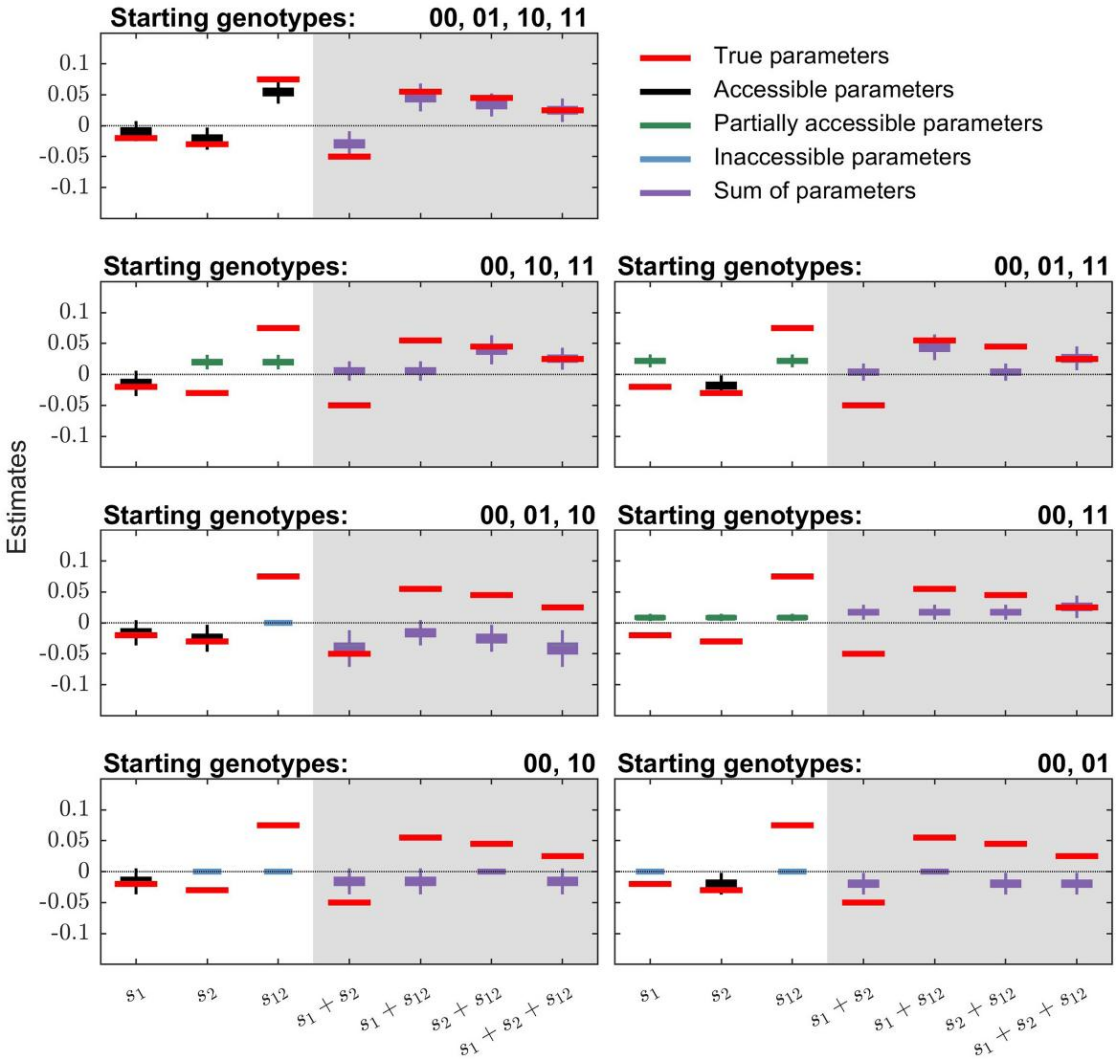
MPL can accurately estimate individual fitness parameters (selection coefficients and epistasis terms) and/or their sums depending on the genetic diversity present in the population. The results are for a two-locus system with positive sign epistasis (selection coefficients $s_{1}=-0.02$, $s_{2}=-0.03$ and pairwise epistasis term $s_{12}=0.075$). All simulation results were computed over 1000 Monte Carlo runs. The boxplots of inferred selection coefficients and epistasis terms are shown on white background in each panel, while those of their sums are shown on gray background. The red bars indicate the true values of the respective terms. The boxplots show the standard data summary (first quartile, median, third quartile) with the whiskers showing 1.5 times the interquartile range. In order to control genetic diversity, both the per locus mutation probability and the per locus recombination probability were set to zero. The population size *N* was set to 1000. The panels depict scenarios with different starting populations. The genotypes contained in the starting population of each simulated scenario are mentioned on top of each panel. The frequency of each non-WT genotype in the initial population was set to 10% of the population size. The sampling parameters were set to $n_{s}=100$, Δt=10, and T=150, where $n_{s}$ is the number of samples, Δt is the time sampling step and *T* is the number of generations used for inference.

We further tested the ability of MPL to infer selection coefficients and epistasis terms under varying degrees of genetic diversity, on a two-locus bi-allelic system, by changing the composition of genotypes in the initial population. We found that the inference of these fitness parameters was quite accurate when all four genotypes appeared at high frequencies in the population, even when both single mutations were deleterious (*top left* panel of fig. 3). When some of the mutant genotypes are never present in the population, however, not all fitness parameters can be accurately inferred (e.g., the *top right* panel of fig. 3). These results show that genetic diversity in data limits which fitness parameters can be inferred.

### Identifiability of Fitness Parameters

Based on patterns of genetic diversity in the time-series data, the estimated fitness parameters can be naturally classified into one of three categories: accessible, partially accessible, or inaccessible, by examining the structure of the integrated covariance matrix used as part of the MPL estimator. Accessible fitness parameters are ones that could be independently estimated in principle (vice-versa for the inaccessible parameters), whereas partially accessible fitness parameters can only be estimated as part of a sum. Specifically, this is done by reducing the integrated covariance matrix to its reduced row-echelon form and checking the linear dependencies of its rows. The fitness parameters whose corresponding rows of the integrated covariance matrix are linearly independent are denoted as accessible. These can be estimated meaningfully. The fitness parameters corresponding to linearly dependent rows are classed as partially accessible. While these parameters cannot be meaningfully estimated individually, we can still estimate their sum. Finally, fitness parameters corresponding to the rows of the integrated covariance matrix with all zero entries are referred to as inaccessible as there is insufficient data to provide a meaningful estimate, either individually or as part of a sum, of these parameters. As an example, we can consider a population with two loci labeled 1 and 2 where only two genotypes are ever observed, one with both WT and one with both mutant alleles. Then the individual coefficients $s_{1},s_{2},s_{12}$ cannot be independently inferred, but their sum $s_{1}+s_{2}+s_{12}$ can be estimated.

When the population consisted of all but one of the single mutant genotypes (*right* and *left* panels of second row of fig. 3), one of the selection coefficients was accessible (and thus accurately inferred) while the remaining two fitness parameters were partially accessible. In scenarios where the double mutant was absent from the population (*left* panel of third row of fig. 3), the selection coefficients were accessible, however there was no data to make any meaningful inference of the epistasis term. When the data contained only the WT and the double mutant genotypes (*right* panel of third row of fig. 3), all three fitness parameters were partially accessible as their inferred sum was accurate even though neither the selection coefficients nor the epistasis terms could be accurately inferred individually. Finally, in scenarios where only one of the two loci was polymorphic, and thus accessible, it was not possible to make a meaningful inference about the selection coefficient at the non-polymorphic locus or the pairwise epistasis term (*bottom left* and *bottom right* panels of fig. 3).

Additional tests demonstrated that the performance of MPL was consistent across a variety of landscapes, comprising of beneficial and/or deleterious selection coefficients and various forms of epistasis like positive, negative, positive sign, and negative sign epistasis (supplementary fig. S1, Supplementary Material online).

### Analysis of a More Complex Five-locus Epistatic Fitness Landscape

**Fig. 4.**
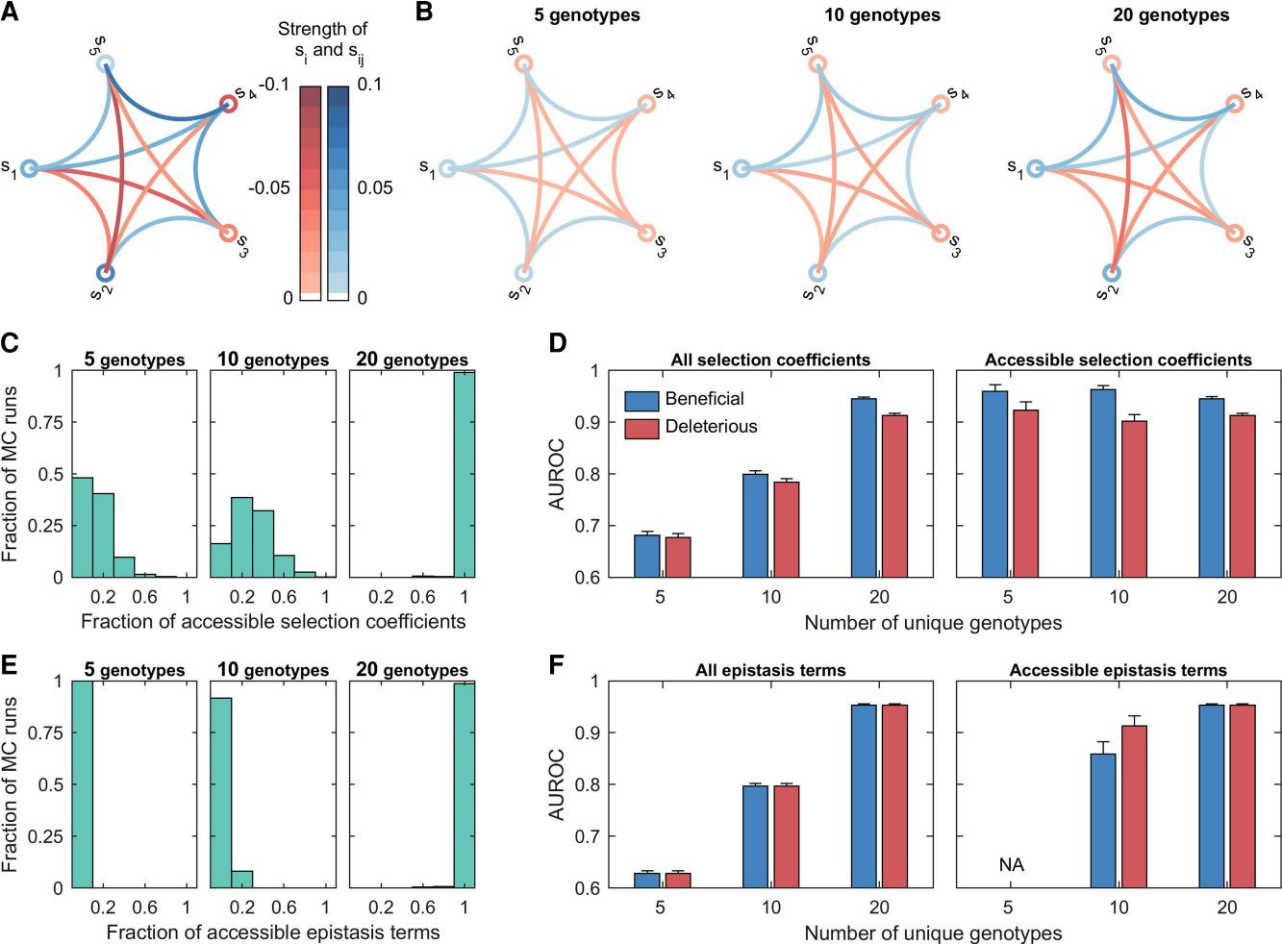
The fraction of selection coefficients and epistasis terms that are accessible depends on the genetic diversity in data. (*A*) shows the true fitness parameters of a five-locus system, where the selection coefficients at loci are shown by circles and pairwise epistasis terms by chords between loci (*blue*: beneficial and *red*: deleterious). Specifically, we set selection coefficients as $s_{1}=0.0385$, $s_{2}=0.0605$, $s_{3}=-0.0318$, $s_{4}=-0.0632$, $s_{5}=0.002$, and epistasis terms as $s_{12}=-0.0361$, $s_{13}=-0.052$, $s_{14}=0.0341$, $s_{15}=0.0262$, $s_{23}=0.0293$, $s_{24}=-0.0278$, $s_{25}=-0.075$, $s_{34}=0.0498$, $s_{35}=-0.0283$, $s_{45}=0.0721$. The *left*, *center*, and *right* panels of (*B*) show the average inferred fitness parameters obtained for different levels of genetic diversity (controlled by varying the number of unique genotypes in the initial population to either 5, 10, or 20). (*C*) shows the fraction of accessible selection coefficients as a function of genetic diversity. The *left* and *right* panels of (*D*) show the mean classification performance computed over all selection coefficients and over only the accessible selection coefficients respectively. The error bars indicate the standard error of the mean. (*E*) shows the fraction of accessible epistasis terms as a function of genetic diversity. The *left* and *right* panels of (*F*) show the classification performance computed over all and only the accessible epistasis terms respectively. “NA” indicates the metric was not computed due to lack of data. The population size *N* was set to 1000. Both the per locus mutation probability and the per locus recombination probability were set to zero in this simulation to control genetic diversity. The frequency of each non-WT genotype in the initial population was set to 5% of the population size. The sampling parameters were set to $n_{s}=100$, Δt=10, and T=100, where $n_{s}$ is the number of samples, Δt is the time sampling step, and *T* is the number of generations used for inference. All simulation results were computed over 1000 Monte Carlo runs.

**Fig. 5.**
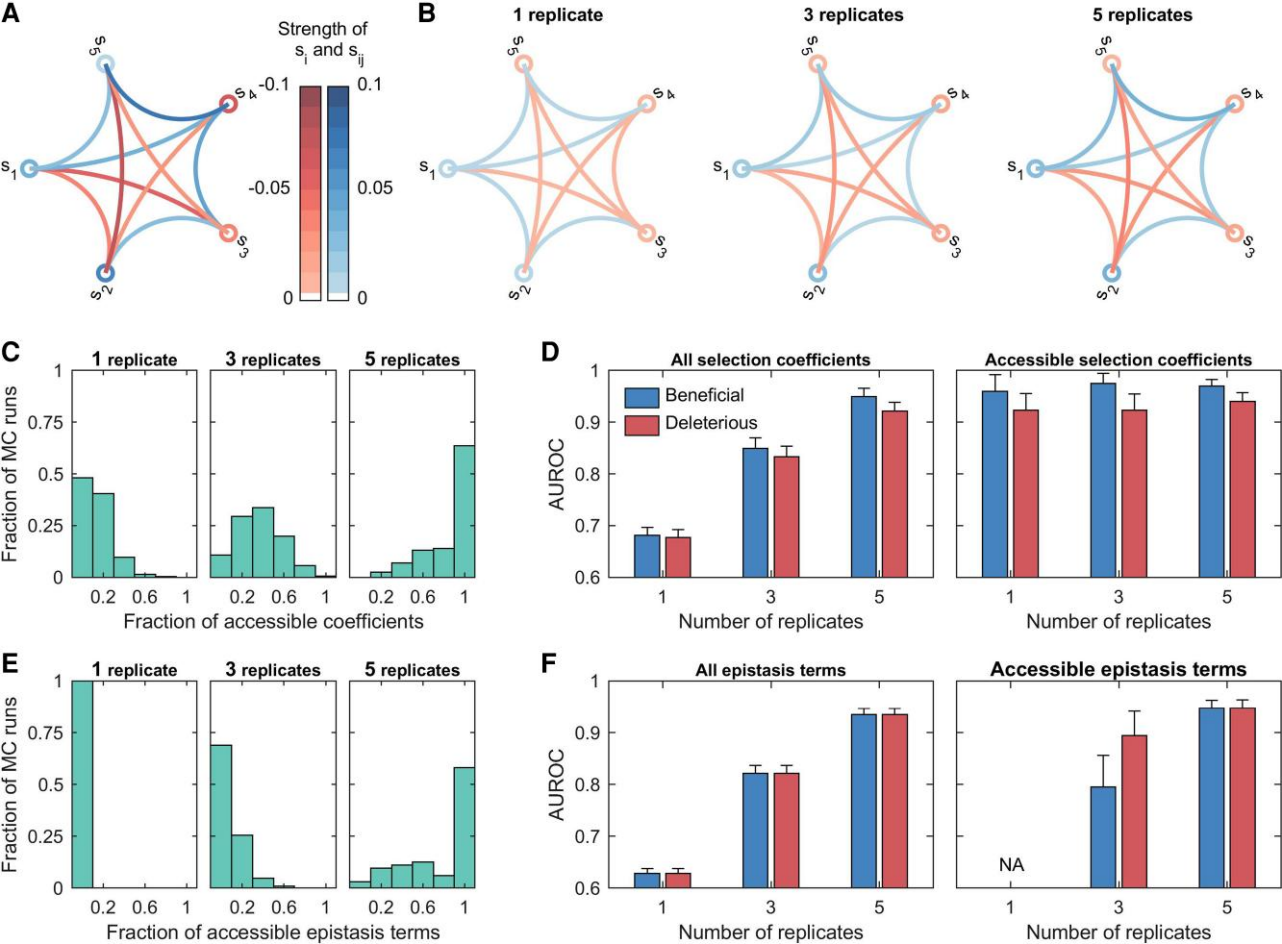
The fraction of selection coefficients and epistasis terms accessible in low genetic diversity scenarios can be increased by combining multiple independent replicates. (*A*) shows the true fitness parameters of a five-locus system, where the selection coefficients at loci are shown by circles and pairwise epistasis terms by chords between loci (*blue*: beneficial and *red*: deleterious). The underlying fitness landscape was the same as in figure 4*A*. The *left*, *center*, and *right* panels of (*B*) show the average inferred fitness parameters obtained for different levels of genetic diversity (controlled by using either 1, 3, or 5 replicates for inference). (*C*) shows the fraction of accessible selection coefficients increases with the increase in genetic diversity. The *left* and *right* panels of (*D*) show the mean classification performance computed over all selection coefficients and over only the accessible selection coefficients respectively. The error bars indicate the standard error of the mean. (*E*) shows the fraction of accessible epistasis terms as a function of genetic diversity. The *left* and *right* panels of (*F*) show the classification performance computed over all and only the accessible epistasis terms respectively. “NA” indicates the metric was not computed due to lack of data. Both the per locus mutation probability and the per locus recombination probability were set to zero in this simulation to control genetic diversity. The population size *N* was set to 1000, and the initial population contained five unique genotypes. The frequency of each non-WT genotype in the initial population was set to 5% of the population size. The sampling parameters were set to $n_{s}=100$, Δt=10, and T=100, where $n_{s}$ is the number of samples, Δt is the time sampling step and *T* is the number of generations used for inference. All simulation results were computed over 1000 Monte Carlo runs.

We ran further simulations on a more complex five-locus system to test the effects of genetic diversity on the inference of MPL. Genetic diversity in these simulations was controlled in two ways: (i) by specifying the number of unique genotypes in the initial population (fig. 4), and (ii) by combining data from multiple independent low genetic diversity replicates (fig. 5).

As expected, there was an increase in the fraction of accessible fitness parameters (figs. 4*C*, 4*E*, 5*C* and 5*E*) and better inference of the fitness landscape (figs. 4*B* and 5*B*) as the level of genetic diversity increased. Our results show that for a given level of genetic diversity, the fraction of accessible selection coefficients is higher than the fraction of accessible epistasis terms (supplementary fig. S2, Supplementary Material online), that is, higher genetic diversity is required for inference of epistasis than that required for inference of selection coefficients alone. This is because, for an epistasis term to be accessible, both corresponding selection coefficients must also be accessible.

We used area under the receiver operating characteristic curve (AUROC) as a performance metric to quantify the ability of MPL to classify beneficial and deleterious fitness parameters. When computed over all selection coefficients (*left* panels of figs. 4*D* and 5*D*) and all pairwise epistasis terms (*left* panels of figs. 4*F* and 5*F*), the results showed higher detection performance with increasing genetic diversity. The poor performance at low genetic diversity was due to the large number of parameters that were either inaccessible or partially accessible, and thus cannot be meaningfully inferred due to lack of data. Computing the AUROC metric but restricted to *only* those selection coefficients classed as accessible revealed that the MPL estimator was able to correctly classify nearly all of such selection coefficients, under all scenarios considered (*right* panels of figs. 4*D* and 5*D*). The classification of accessible epistasis terms also showed good performance at moderate and high genetic diversity (*right* panels of figs. 4*F* and 5*F*). Although none of the epistasis terms were accessible at low genetic diversity, combining multiple replicates using (22) resulted in some epistasis terms becoming accessible (fig. 5*E*).

Similar results were obtained across a range of fitness landscapes differing in the degree of sparsity in their pairwise epistasis terms (supplementary fig. S3, Supplementary Material online). These tests demonstrate that MPL has a very good ability to detect those fitness parameters for which there is sufficient data to enable inference and classification.

### Robustness to Sampling Parameters

The accuracy of the estimator depends on how well the underlying population dynamics is sampled. This includes how often the population is sampled in time, the number of samples measured at each time point, and the number of generations used for inference. Here we test the robustness of the MPL method with respect to these sampling parameters. In general, one would expect performance to degrade as samples are taken further apart in time (increasing time sampling step Δt) for a fixed number of generations used for inference, *T*, or as the number of generations used for inference is reduced for a fixed time sampling step, as less of the trajectory dynamics are captured in both these sampling scenarios. Moreover, taking limited samples at each time point would reduce the accuracy of the allele frequency estimates which may also compromise the accuracy of the MPL estimate.

**Fig. 6.**
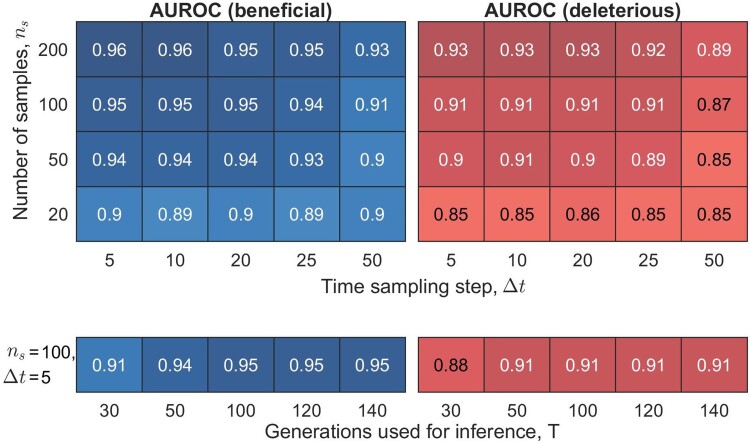
MPL is robust to variation in sampling parameters. The *top left* and *top right* panels show the mean AUROC performance of detecting accessible beneficial and deleterious selection coefficients, respectively. The *top* panels show mean AUROC performance for a range of values of number of samples, $n_{s}$, and time sampling step, Δt, with a fixed value of number of generations used for inference, T=100, while the *bottom* panels show the performance for a range of values of *T* with $n_{s}=100$ and Δt=5. Results are for a five-locus system with the fitness landscape shown in figure 4*A*. The population size *N* was set to 1000 and the initial population contained 20 unique genotypes. Other simulation parameters included per locus mutation probability $\mu =10^{-4}$ and per locus recombination probability $r=10^{-4}$. All results were averaged over 1000 Monte Carlo runs.

To test the robustness of the estimator, we ran extensive simulations under various sampling conditions. These simulations demonstrated that MPL can accurately detect both accessible selection coefficients and accessible epistasis terms for a range of sampling parameters (fig. 6 and supplementary fig. S4, Supplementary Material online, respectively). MPL performed quite well even when the observed data consisted of a low number of samples, $n_{s}$, with only a few time samples (large time sampling step, Δt). For example, at $n_{s}=50$ (from a population of N=1000), the AUROC of detecting accessible beneficial selection coefficients (*top left* panel of fig. 6) varied from 0.94 to 0.9 when the time sampling step was increased from Δt=5 to Δt=50 (corresponding to 21 and 3 time samples, respectively ,over T=100 generations used for inference). Similarly, MPL performed well even when only a few time points that captured the evolutionary dynamics were used for inference. For example, the AUROC of detecting accessible beneficial selection coefficients (*bottom left* panel of fig. 6) varied from 0.91 to 0.95 when the number of generations used for inference was increased from T=30 to T=140 (corresponding to 7 and 29 time points, respectively, with Δt=5). These results show that MPL estimator is robust to reasonable limitations in sampling depth and frequency.

### Comparison with Other Models

#### A Model that Does not Account for Epistasis

For fitness landscapes with epistasis, any inference model that does not explicitly account for epistasis will ascribe the effect of epistasis terms to individual selection coefficients, thereby over- or under-estimating them. To test this, we ran simulations to compare the performance of the MPL method, which accounts for both linkage and epistasis, with the one we proposed previously, which accounts only for linkage and considers a first-order fitness model with no epistasis (Sohail et al. 2021). Here we term this variant as “MPL (without epistasis)”. Simulations on simple two-locus systems with different fitness parameter settings showed that when the epistasis term was accessible (based on genetic diversity in the data), MPL estimates were more accurate than MPL (without epistasis) for scenarios where the fitness landscape had epistasis, particularly when any pair of fitness parameters had opposite signs (supplementary fig. S5, Supplementary Material online).

**Fig. 7.**
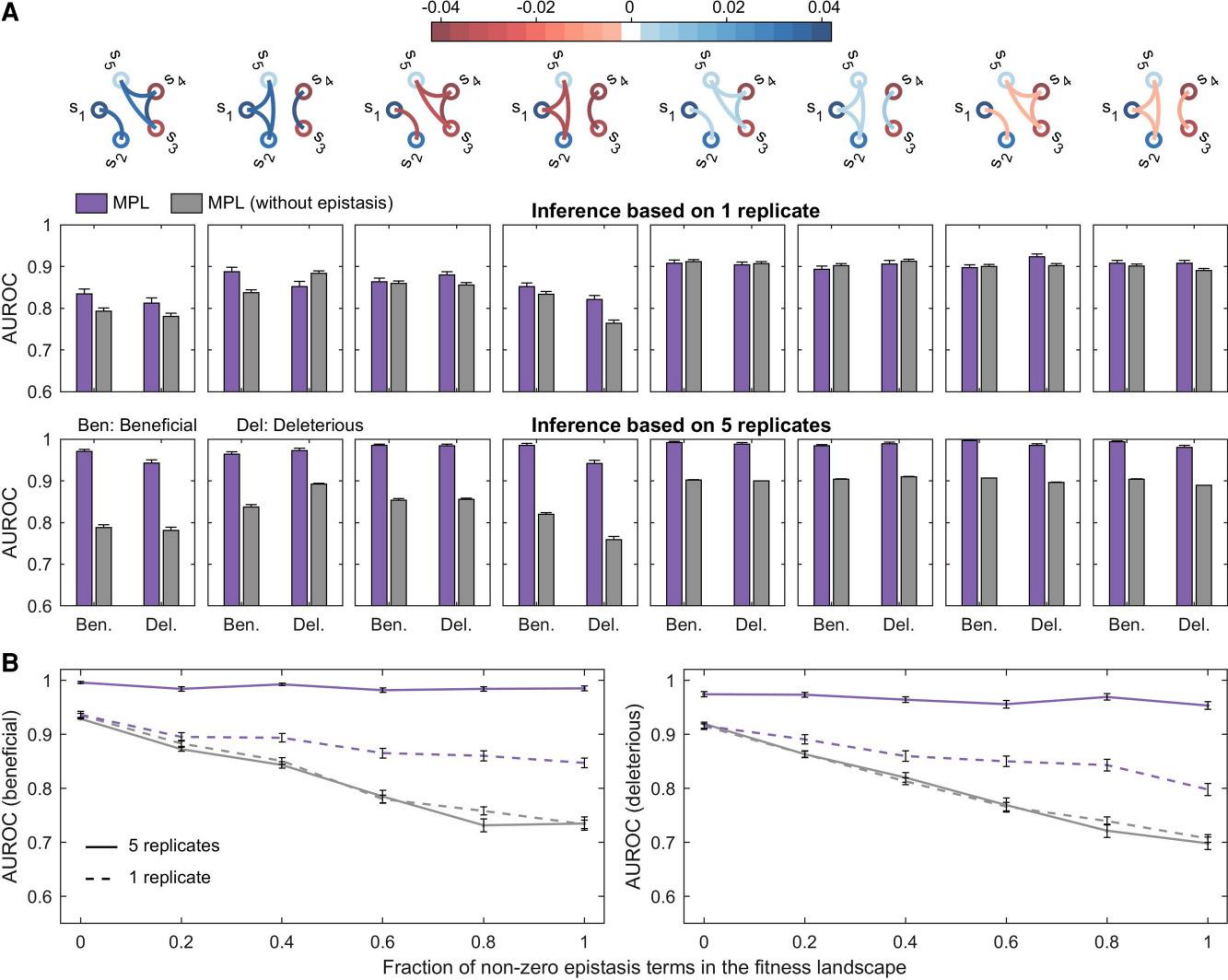
Ability of MPL to accurately identify selection coefficients is robust to the density and the strength of non-zero epistasis terms in the fitness landscape. (*A*) panels in the *top* row show simple fitness landscapes, that is, no epistasis links between loci with mutant allele selection coefficients of opposite signs and all epistasis links of similar strengths and the same sign, while panels in the *center* and *bottom* rows show the classification performance of MPL and MPL (without epistasis) on data consisting of a single low genetic diversity replicate and that where five low-diversity replicates are combined, respectively. (*B*) shows the AUROC performance of both methods for varying density of epistasis terms in the fitness landscape under high and low genetic diversity scenarios. Error bars indicate the standard error of the mean. All fitness landscapes had two beneficial, two deleterious, and one neutral selection coefficients. Both the selection coefficients and epistasis terms, in the fitness landscapes with strong epistasis in (*A*), were randomly drawn from uniform distributions over the ranges [0.03,0.04] and [−0.03,−0.04] for beneficial and deleterious fitness parameters, respectively. While, the selection coefficients of the fitness landscapes with weak epistasis in (*A*) were drawn from the same distributions as before but the epistasis terms, positive and negative, were drawn from uniform distributions over the ranges [0.003,0.004] and [−0.003,−0.004], respectively. For the fully connected fitness landscape in (*B*), we used the same fitness landscape as in figure 4*A*. Half of the epistasis terms in this fitness landscape were positive while the other half were negative. To obtain a fitness landscape with a desired sparsity level, we set a randomly selected set of epistasis terms to zero. For a given sparsity level, we averaged the performance results over 10 randomly selected landscapes, except for the fully connected and the purely additive (all epistasis terms set to zero) landscape cases where the results are for a single landscape. Numerical values of all fitness landscapes used in these simulations are provided in supplementary table S1, Supplementary Material online. The initial population contained 5 unique genotypes, with per locus mutation probability $\mu =10^{-4}$ and per locus recombination probability $r=10^{-4}$, and population size N=1000. The sampling parameters were set to $n_{s}=100$ and Δt=10, with T=100 generation used for inference. All simulation results were computed over 1000 Monte Carlo runs.

Next, we tested the classification performance of the two methods in a five-locus system. Initially, we chose relatively simple structures for the fitness landscapes; that is, no epistasis links between loci with mutant allele selection coefficients of opposite signs, and all epistasis links having similar strengths and the same sign (*top* row panels of fig. 7*A*). We also varied the strength of the epistasis terms from strong (both selection coefficients and epistasis terms drawn from the same distribution) to weak (epistasis terms an order of magnitude weaker than the selection coefficients). Our results showed that when the genetic diversity in the data was low, the relative classification performance of the two methods was dependent on the underlying fitness landscape (*middle* row panels of fig. 7*A*), with MPL generally performing better than or as well as MPL (without epistasis). However, combining multiple low-diversity independent replicates using (22) resulted in MPL performing significantly better than MPL (without epistasis) in all scenarios tested, including weak, strong, positive, and negative epistasis (*bottom* row panels of fig. 7*A*).

We also compared the performance of the two methods on more complicated fitness landscapes with both positive and negative epistasis terms, and on fitness landscapes of varying density of non-zero epistasis terms (i.e., different epistasis sparsity levels). We generated several such fitness landscapes, with similar magnitudes of selection coefficients and pairwise epistasis terms as the fitness landscape in figure 4*A*, but differing in the density of epistasis terms, ranging from a purely additive landscape (no epistasis terms) to a highly epistatic landscape (with all pairwise epistasis terms being non-zero). We grouped these landscapes on the basis of number of non-zero pairwise epistasis terms. Our results demonstrated that in high genetic diversity scenarios, MPL had better performance than MPL (without epistasis) regardless of the density of epistasis terms in the fitness landscape (fig. 7*B*). In scenarios where the genetic diversity in the data was low, the two methods had similar performance when the underlying fitness landscape was additive or had low density of pairwise epistasis terms, while MPL had superior performance when the fitness landscape was highly epistatic (fig. 7*B*). Interestingly, our simulations showed that even in scenarios where none of the epistasis terms were accessible (supplementary fig. S2, Supplementary Material online), MPL still showed a marked improvement in performance over MPL (without epistasis) in classifying accessible selection coefficients (supplementary fig. S6, Supplementary Material online). Overall, our approach enabled us to disentangle the confounding effects of linkage and epistasis from data, resulting in more accurate inference of fitness parameters.

#### A Model that Accounts for Epistasis

We further compared our method with that of Illingworth et al. (2014) (see supplementary text, Supplementary Material online), a state-of-the-art method that also accounts for epistasis, but unlike MPL, is based on a deterministic evolutionary model, and requires the use of numerical optimization algorithms. Our simulations demonstrated that MPL showed better classification performance and was considerably faster (supplementary fig. S7, Supplementary Material online). The performance improvement of MPL was particularly evident for scenarios with deleterious selection coefficients and with negative sign epistasis (supplementary fig. S8, Supplementary Material online).

### Scaling to Larger Systems

For a bi-allelic system, the number of selection coefficients grow linearly with the number of loci in the system, *L*, while the number of epistasis terms grow quadratically as L(L−1)/2. To test the effect this increase in the number of fitness parameters has on their accessibility and the performance of MPL, we simulated systems of different sizes (10-, 20-, and 30-locus systems). Our results showed that the fraction of accessible parameters and the AUROC classification performance tended to reduce as the number of loci increased. However, by increasing the genetic diversity through multiple replicate combining, all fitness parameters eventually became accessible for all three system sizes (*top* row of supplementary fig. S9, Supplementary Material online). Simulations also showed that the accessibility of fitness parameters was robust to the level of sparsity in the underlying fitness landscape (supplementary fig. S10, Supplementary Material online). Consistent with earlier results, increased genetic diversity for a given system size resulted in improved classification performance (supplementary fig. S9, Supplementary Material online).

### Computational Complexity

The closed-form nature of the MPL estimate (21) makes it potentially computationally efficient. The two most computationally intensive steps in the algorithm are (i) calculating the triple and quadruple mutant allele frequencies from the data, and (ii) inversion of the regularized integrated covariance matrix. The number of triple and quadruple mutant frequencies required for computing the inverse term in (21) increases as $L^{4}$, where *L* is the number of loci. However, this number can be reduced based on the genetic diversity in the data. For instance, for any locus-pair (i,j) whose double mutant frequency is zero, it follows that any three tuple (i,j,k) involving the same pair will have a triple mutant frequency of zero and hence its calculation can be avoided. Similarly, the number of quadruple mutant frequencies that need to be computed can also be reduced. The computations required for computing the inverse term can also be reduced by considering only the polymorphic loci $L_{p}<L$, instead of the whole sequence, leading to $R_{p}=L_{p}(L_{p}+1)/2$ parameters to be estimated. The inverse would then require $O(R_{p}^{3})$ computations, with $R_{p}\ll R$ in practice for realistic data sets.

## Discussion

Epistasis is a pervasive phenomenon that can strongly shape the evolution. Genetic time-series data provide an opportunity to detect and estimate epistatic contributions to fitness. However, developing methods that can efficiently yield accurate inferences has remained a challenge. Here we proposed a method to address this challenge. Our approach is a physics-based approach that builds upon a framework that we recently introduced for non-epistatic models (Sohail et al. 2021). Through simulations, we demonstrated that our method can accurately infer both pairwise epistasis effects and individual selection coefficients, provided sufficient variation exists in the data. Moreover, the method systematically reveals necessary conditions on genetic variation in the data in order for accurate inferences to be possible, and for the separate contributions of epistasis and allele selection coefficients to be inferrable.

MPL uses a path integral to approximate the likelihood of a set of evolutionary parameters (including epistasis), given an observed time-series of allele frequencies and their correlations. This framework can also be adapted for different evolutionary scenarios. In recent work, it was applied together with epidemiological models to infer the transmission effects of mutations from genomic surveillance data, and to study the evolution of SARS-CoV-2 (Lee et al. unpublished data).

The data input to MPL, under a fitness model with pairwise epistasis terms, consists of the single, double, triple, and quadruple mutant allele frequencies. While these are readily available from long-read sequencing data, the double and higher mutant allele frequencies cannot be computed extensively for short-read data. More work is required to develop methods that can accurately detect or infer selection and epistasis for such data sets. However, the trend toward longer read lengths in third-generation sequencing technologies (Pollard et al. 2018) suggests that higher-order mutant frequencies will be more readily available in future data sets. While fitness models with higher-order epistasis involving more than two mutant alleles are also possible (Weinreich et al. 2013), here we restricted our analysis to a fitness model with pairwise epistasis terms. In principle, the MPL framework can be extended to account for higher-order epistasis terms by explicitly modeling the evolution of higher-order mutant allele frequencies. However, the contribution of epistasis terms to fitness typically declines with their order (Weinreich et al. 2018) and, at least in some scenarios, the gain achieved by modeling higher-order epistasis beyond pairwise terms appear to be minimal (Lozovsky et al. 2021).

MPL, like all inference methods, requires sufficient diversity to enable parameter inference. For a fitness model with pairwise epistasis terms, the number of model parameters to be inferred increases quadratically with the sequence length. As such, data with insufficient variation may lead to a situation where most of the model parameters are partially accessible or inaccessible (supplementary fig. S2, Supplementary Material online). This is not intrinsically a limitation of our specific method, but rather of a lack of exploratory power in the data. However, in scenarios where multiple of such low-diversity independent replicates are available, MPL offers a solution to overcome this limitation by providing a systematic way to combine low-diversity replicates.

The current approach infers a fitness landscape with epistasis terms between every pair of mutant alleles, in contrast to an additive fitness landscape inferred in Sohail et al. (2021). One can also consider selecting the most likely fitness model, given the data, from a reduced set of models with different densities of epistasis terms using a model selection approach. However, it may only be feasible to pursue model selection approaches for moderate sized systems due to the exponential increase in the number of possible models with increasing system size. An alternative approach can be to apply a sparsity constraint on the epistasis terms. Future work on this problem can leverage sparsity inducing techniques such as the least absolute shrinkage and selection operator (LASSO) regression family of methods (Tibshirani 1996; Yuan and Lin 2006), to come up with a computationally efficient algorithm suitable for systems with hundreds or thousands of segregating mutations.

## Materials and Methods

### Model

We consider a population of *N* individuals evolving under a WF model with selection, mutation and recombination. Each individual is represented by a sequence of length *L*. The loci are assumed to be bi-allelic where each locus is either 0 (wild-type (WT)) or 1 (mutant), thus resulting in $M=2^{L}$ genotypes. We consider a fitness model that accounts for epistasis arising due to pairwise interactions between alleles at different loci. The Wrightian fitness $f_{a}$ of the *a*th genotype can then be written as

(5) $$f_{a}=1+\sum_{i=1}^{L}s_{i}g_{i}^{a}+\sum_{i=1}^{L}\sum_{\;j=i+1}^{L}s_{ij}g_{i}^{a}g_{j}^{a},$$

where $s_{i}$ and $s_{ij}$ denote the time-invariant selection coefficients and pairwise epistasis terms respectively, and $g_{i}^{a}$ represents the allele (either 0 or 1) at the *i*th locus of the *a*th genotype. The population is completely specified by the M×1 genotype frequency vector $z(t)=(z_{1}(t),\ldots ,z_{M}(t))$, where $z_{a}(t)=n_{a}(t)/N$ and $n_{a}(t)$ denotes the number of individuals in the population that belong to genotype *a* at generation *t*.

Under WF dynamics, the probability of observing genotype frequencies z(t+1) at generation t+1, given genotype frequencies of z(t) at generation *t* is

(6) $$P(z(t+1)\;|\;z(t))=N!\prod_{a=1}^{M}\frac{(p_{a}(z(t)){)}^{Nz_{a}(t+1)}}{(Nz_{a}(t+1))!}$$

with

(7) $$p_{a}(z(t))=\frac{y_{a}(t)f_{a}+\sum_{b\neq a}(\mu_{ba}y_{b}(t)f_{b}-\mu_{ab}y_{a}(t)f_{a})}{\sum_{b=1}^{M}y_{b}(t)f_{b}}.$$

Here $\mu_{ba}$ is the probability of genotype *b* mutating to genotype *a*, and $y_{a}(t)$ is the frequency of genotype *a* after recombination

(8) $$y_{a}(t)=(1-r{)}^{L-1}z_{a}(t)+(1-(1-r{)}^{L-1})\psi_{a}(z(t)),$$

where *r* is the recombination probability per locus per generation and $\psi_{a}(z(t))$ is the probability that a recombination of two individuals results in an individual of genotype *a* (see supplementary text, Supplementary Material online for details).

We assume the genotype frequencies are observed at non-consecutive generations $t_{k}$, with k∈{0,1,…,K}. Then, the probability that the genotype frequency vector follows a particular evolutionary path $\left(z(t_{1}),z(t_{2}),\ldots ,z(t_{K})\right)$, conditioned on the initial state $z(t_{0})$, is

(9) $$P((z(t_{k}){)}_{k=1}^{K}\;|\;z(t_{0}))=\prod_{k=0}^{K-1}P(z(t_{k+1})\;|\;z(t_{k})).$$

This expression can be used to infer evolutionary parameters. However, the inference problem is difficult due to the intractability of the fractional form of (7). Following the approach used in Sohail et al. (2021), we simplify the inference problem using a path integral. This allows us to obtain closed-form estimates of selection coefficients and epistasis terms. Even though the WF dynamics is defined at the genotype level (9), here we develop its simplified allele-level version for transparency. We show later in this section that both the genotype and allele-level analyses lead to the same expression for the estimate of fitness parameters. For ease of exposition, we assume here that the probability of mutating from a WT to mutant allele is the same as that from mutant allele to WT, which we denote by μ. However, this assumption can be easily relaxed (see supplementary text, Supplementary Material online for details where we derive the estimator with asymmetrical mutation probabilities).

#### Linear Mapping between Genotype and Allele Frequencies

The allele frequencies can be described by taking a linear combination of genotype frequencies. Specifically,

(10) $$\begin{array}{rl} x_{i}(t) & =\sum_{a=1}^{M}g_{i}^{a}z_{a}(t),\;x_{ij}(t)=\sum_{a=1}^{M}g_{i}^{a}g_{j}^{a}z_{a}(t),\\ x_{ijk}(t) & =\sum_{a=1}^{M}g_{i}^{a}g_{j}^{a}g_{k}^{a}z_{a}(t),\;x_{ijkl}(t)=\sum_{a=1}^{M}g_{i}^{a}g_{j}^{a}g_{k}^{a}g_{l}^{a}z_{a}(t), \end{array}$$

where $x_{i}(t)$, $x_{ij}(t)$, $x_{ijk}(t)$, and $x_{ijkl}(t)$ are the single, double, triple, and quadruple mutant allele frequencies at locus *i*, locus-pair (i,j), locus-triplet (i,j,k), and locus-quartet (i,j,k,l), respectively, at generation *t*.

We will explicitly model the evolution of the single and double mutant allele frequencies, which we represent by the single vector,

(11) $$x(t)=(x_{1}(t),\ldots ,x_{L}(t),x_{12}(t),x_{13}(t),\ldots ,x_{(L-1)L}(t)).$$

For notational convenience (to facilitate sequential indexing), we equivalently write

(12) $$x(t)=(x_{1}(t),\ldots ,x_{L}(t),x_{L+1}(t),\ldots ,x_{R}(t)),$$

where R=L(L+1)/2. Here, and in the following, we differentiate between non-italic and italic scalar notation. From (11) and (12), we have $x_{e}(t)=x_{i}(t)$ for e≤L, and $x_{e}(t)=x_{ij}(t)$ for L<e≤R. We will explicitly denote the index mapping as e↦i for the former case, and e↦(i,j) for the latter.

#### Path Integral

We model the evolution of both the single and double mutant allele frequencies. In the allele-level path integral, these are required to obtain estimates of the selection coefficients and the pairwise epistasis terms (see supplementary text, Supplementary Material online for the genotype-level path integral formulation).

The probability of observing a path of allele frequencies $\left(x(t_{1}),x(t_{2}),\ldots ,x(t_{K})\right)$ conditioned on $x(t_{0})$ is given by

(13) $$P((x(t_{k}){)}_{k=1}^{K}\;|\;x(t_{0}))=\prod_{k=0}^{K-1}P(x(t_{k+1})\;|\;x(t_{k})).$$

We use a path integral to approximate this probability, as described in New Approaches. This gives the following closed-form approximation of the transition probability (see supplementary text, Supplementary Material online for details)

(14) $$P(x(t_{k+1})\;|\;x(t_{k}))\approx \phi (x(t_{k+1})\;|\;x(t_{k}))\prod_{e=1}^{R}dx_{e}(t_{k+1}),$$

where

$$\phi (x(t_{k+1})\;|\;x(t_{k}))={\left(\frac{N}{2\pi \Delta t_{k}}\right)}^{R/2}\frac{\exp\;(-(N/2)\Theta (x(t_{k+1}),x(t_{k})))}{\sqrt{\text{det}C(x(t_{k}))}}$$

with $\Delta t_{k}=t_{k+1}-t_{k}$ and

$$\begin{array}{rl} & \Theta (x(t_{k+1}),x(t_{k}))\\ & \;=\frac{1}{\Delta t_{k}}\sum_{e=1}^{R}\sum_{\;f=1}^{R}[x_{e}(t_{k+1})-(x_{e}(t_{k})+d_{e}(x(t_{k}))\Delta t_{k})]\\ & \;\times (C^{-1}(x(t_{k})){)}_{ef}[x_{f}(t_{k+1})-(x_{f}(t_{k})+d_{f}(x(t_{k}))\Delta t_{k})] \end{array}$$

Here $d_{e}(x(t_{k}))$ describes the expected change in allele frequencies (either single mutant or pairwise, depending on *e*) from generation $t_{k}$ to $t_{k+1}$, given by

(15) $$\begin{array}{rl} d_{e}(x(t_{k})) & =x_{e}(t_{k})(1-x_{e}(t_{k}))s_{e}+\sum_{\;f\neq e}C_{ef}(x(t_{k}))s_{f}\\ & \;+\mu v_{e}(x(t_{k}))+r\;\eta_{e}(x(t_{k})). \end{array}$$

Above, $s=(s_{1},\ldots ,s_{L},s_{12},s_{13},\ldots ,s_{(L-1)L})$ is the vector of selection coefficients and pairwise epistasis terms, with terms having the corresponding non-italic representation $s_{e}$, defined analogously to (12). We have also defined

$$v_{e}(x(t_{k}))=\left\{ \begin{array}{ll} 1-2x_{i}(t_{k}) & 1\leq e\leq L\\ x_{i}(t_{k})+x_{\;j}(t_{k})-4x_{ij}(t_{k}) & L<e\leq R, \end{array}\right.$$

and

$$\eta_{e}(x(t_{k}))=\left\{ \begin{array}{ll} 0 & 1\leq e\leq L\\ \left(i-j)(x_{ij}(t_{k})-x_{i}(t_{k})x_{\;j}(t_{k})\right) & L<e\leq R, \end{array}\right.$$

where e↦i for e≤L and e↦(i,j) for L<e≤R. The first term in (15) represents the expected change in allele frequencies (either single mutant or pairwise, depending on *e*) due to *e*th fitness parameter, the second term represents the change due to all but the *e*th fitness parameter, the third term in (15) represents the contribution due to net mutational flow, while the fourth term represents the contributions to the expected change in allele frequencies due to recombination.

The matrix $C(x(t_{k}))$ is a symmetric R×R matrix describing the covariances of the allele frequencies at generation $t_{k}$. This can be partitioned into four sub-matrices, each with an intuitive interpretation (details in supplementary text, Supplementary Material online). Briefly, for e≤L and f≤L, with mapping e↦i and f↦j, the elements

(16) $$C_{ef}(x(t_{k}))=x_{ij}(t_{k})-x_{i}(t_{k})x_{j}(t_{k})$$

are the covariance between mutants at loci *i* and *j*; for e≤L and L<f≤R, with mapping e↦i and f↦(j,k), the elements

(17) $$C_{ef}(x(t_{k}))=x_{ijk}(t_{k})-x_{i}(t_{k})x_{\;jk}(t_{k})$$

are the covariance between mutant at locus *i* and double mutant at locus-pair (j,k); the elements of $C(x(t_{k}))$ for L<e≤R and f≤L are the same as (17) due to the symmetric nature of the covariance matrix; while for L<e≤R and L<f≤R, with mapping e↦(i,j) and f↦(k,l), the elements

(18) $$C_{ef}(x(t_{k}))=x_{ijkl}(t_{k})-x_{ij}(t_{k})x_{kl}(t_{k})$$

are the covariance between the double mutants at locus-pair (i,j) and double mutants at locus-pair (k,l).

Substituting (14) in (13) gives an approximation for the probability of the single and pairwise mutant allele frequencies following the evolutionary path $x(t_{1}),x(t_{2}),\ldots ,x(t_{K})$, conditioned on $x(t_{0})$.

### Marginal Path Likelihood Estimator with Epistasis

The MPL parameter estimates are obtained by adopting a Bayesian approach. Specifically, we use the maximum a posteriori (MAP) criterion to find the most likely selection coefficients and epistasis terms given the measured single, double, triple, and quadruple mutant frequencies at each sampling time point, along with knowledge of evolutionary parameters *N*, μ, and *r*. For the purpose of developing an efficient inference approach, we assume that the observed frequencies are equal to the true frequencies in the population. The MPL estimate of the selection coefficients and epistasis terms can thus be obtained by solving

(19) $$\hat{s}=\underset{s}{\arg\;\max}\;L(s;N,r,\mu ,(x(t_{k}){)}_{k=0}^{K})P^{\mathrm{prior}}(s),$$

where $P^{\mathrm{prior}}(s)$ is the assumed (conjugate) prior

$$P^{\mathrm{prior}}(s)=\frac{1}{(2\pi \sigma^{2}{)}^{R/2}}\exp\;\left(-\frac{1}{2\sigma^{2}}s^{T}s\right),$$

with mean zero and variance $\sigma^{2}>0$, and the likelihood of the selection coefficients and epistasis terms, s, given the observed data can be expressed as

(20) $$\begin{array}{rl} L(s;N,r,\mu ,(x(t_{k}){)}_{k=0}^{K}) & =P((x(t_{k}){)}_{k=1}^{K}\;|\;x(t_{0}),N,r,\mu ,s)\\ & =\prod_{k=0}^{K-1}P(x(t_{k+1})\;|\;x(t_{k}),N,r,\mu ,s). \end{array}$$

While it is challenging to calculate the likelihood (20) exactly, the task is simplified by using the path integral approach outlined in the previous section with some modifications (see supplementary text, Supplementary Material online for details) to account for time-samples drawn from non-unit time intervals, $\Delta t_{k}=t_{k+1}-t_{k}$. Following this approach, the MAP solution is evaluated as

(21) $$\begin{array}{rl} {\hat{s}}_{e} & =\sum_{\;f=1}^{R}{\left[\sum_{k=0}^{K-1}\Delta t_{k}C(x(t_{k}))+\gamma I\right]}_{ef}^{-1}\\ & \;\times \left[x_{f}(t_{K})-x_{f}(t_{0})-\mu \sum_{k=0}^{K-1}\Delta t_{k}v_{f}(x(t_{k})\right)\\ & \;-r\sum_{k=0}^{K-1}\Delta t_{k}\eta_{f}(x(t_{k}))], \end{array}$$

for e=1,…,R. The inverse term consists of the covariance matrix of single and double mutant allele frequencies (a function of single, double, triple, and quadruple mutant allele frequencies) integrated over time, which we refer to as the *integrated covariance matrix*, plus a regularization term, where $\gamma =1/N\sigma^{2}$ and *I* is the identity matrix.

The MPL estimator (21) has an intuitive interpretation. It computes the observed change in the single and double mutant allele frequencies between the final and the initial time points, adjusts it by accounting for the (inward and outward) mutational flows of single and double mutant frequencies over time, and then applies a correction to the double mutant frequencies to account for the effect of recombination. Finally, it accounts for linkage effects through the inverse of the (regularized) integrated covariance matrix.

As shown in (21), significant changes in mutant frequencies—that is, ones that are substantially larger than those expected due to finite population size alone—that cannot readily be explained by mutation, recombination, or the effects of background mutations provide evidence of selection or epistatic interactions. For example, mutant alleles that are separated by a long distance on the genome and which remain strongly linked despite recombination would be evidence of a positive epistatic interaction.

### Combining Multiple Independent Observations

The approach naturally lends itself to incorporating data from multiple replicates. These replicates may represent independent evolutionary paths with possibly distinct sampling parameters and starting conditions. Let $t_{1}^{q},\ldots ,t_{K_{q}}^{q}$ be the sampling times of the *q*th replicate and $x_{i}^{q}(t_{k}^{q})$, $x_{ij}^{q}(t_{k}^{q})$ be the single and double mutant allele frequencies at the *i*th locus and the (i,j)th locus-pair, respectively, at generation $t_{k}^{q}$. The observed trajectory of the single and double mutant allele frequencies of the *q*th replicate is thus denoted as $x^{q}(t_{k}^{q})=(x_{1}^{q}(t_{k}^{q}),\ldots ,x_{L}^{q}(t_{k}^{q}),x_{12}^{q}(t_{k}^{q}),x_{13}^{q}(t_{k}^{q}),\ldots ,x_{(L-1)L}^{q}(t_{k}^{q}))$. The MPL estimate in this case is given as (see supplementary text, Supplementary Material online for details)

(22) $$\begin{array}{rl} {\hat{s}}_{e} & =\sum_{\;f=1}^{R}{\left[\sum_{q=1}^{Q}\sum_{k=0}^{K_{q}-1}\Delta t_{k}^{q}C(x^{q}(t_{k}^{q}))+\gamma I\right]}_{ef}^{-1}\\ & \;\times \sum_{q=1}^{Q}\left(x_{f}^{q}(t_{K_{q}}^{q})-x_{f}^{q}(t_{0}^{q})-\mu \sum_{k=0}^{K_{q}-1}\Delta t_{k}^{q}v_{f}(x^{q}(t_{k}^{q})\right)\\ & \;-r\sum_{k=0}^{K_{q}-1}\Delta t_{k}^{q}\eta_{f}(x^{q}(t_{k}^{q}))), \end{array}$$

where *Q* is the number of replicates being combined, $\Delta t_{k}^{q}=t_{k+1}^{q}-t_{k}^{q}$, $\gamma =1/N\sigma^{2}$ as before, and $C(x^{q}(t_{k}^{q}))$ is the covariance matrix of the mutant allele frequencies at generation $t_{k}^{q}$ for the *q*th replicate.

### Equivalence with the Genotype Estimate

The MPL estimate above was derived using a path integral for the mutant allele frequencies even though the WF evolutionary process is defined at the genotype level. One may ask if working at the level of allele frequencies leads to some loss in optimality? To check this, we derive the MPL estimate of the selection coefficients and epistasis terms directly from the genotype path-likelihood (see supplementary text, Supplementary Material online for details), in contrast to the mutant allele path-likelihood (20) as was done above. For the observed path of the genotype frequencies $\left(z(t_{0}),z(t_{1}),\ldots ,z(t_{K})\right)$, the MPL estimate is obtained by solving

(23) $$\hat{s}=\underset{s}{\arg\;\max}L(s;N,\mu ,(z(t_{k}){)}_{k=0}^{K})P^{\mathrm{prior}}(s),$$

where

(24) $$\begin{array}{rl} L(s;N,\mu ,(z(t_{k}){)}_{k=0}^{K}) & =P((z(t_{k}){)}_{k=1}^{K}\;|\;z(t_{0}),N,\mu ,s)\\ & =\prod_{k=0}^{K-1}P\left(z(t_{k+1})\;|\;z(t_{k}),N,\mu ,s\right). \end{array}$$

We obtain the same expression for the MPL estimate (21) by solving (23) as shown in the supplementary text, Supplementary Material online, that is, there is no loss in optimality by working with the marginal allele frequencies. This implies that knowledge of up to fourth-order allele frequencies is sufficient to estimate selection coefficients and pairwise epistatic interactions. At least within the diffusion approximation, higher order frequencies do not carry additional information needed to estimate the fitness effects of individual mutations or pairwise epistasis.

### Simulation Setup

We generated evolutionary histories by running WF simulations, with selection, mutation, and recombination, consisting of a population of *N* bi-allelic sequences evolving for *T* generations. We then randomly sampled $n_{s}$ sequences every Δt generations, and used these sampled trajectories for inference of fitness parameters. The specific values of these parameters used in simulations are specified in the figure captions.

In simulations where it was required to control genetic diversity in a population, we specified the number and the frequencies of the unique genotypes in the initial population, and disallowed mutations and recombination. We refer to the all-zero genotype as the WT genotype. In simulations where the initial population contained more than one unique genotype, one of these was always the WT while the others were chosen from the set of remaining $2^{L}-1$ possible genotypes at random, without replacement. All simulation results were computed over 1000 Monte Carlo runs. Unless stated otherwise, the initial frequency of each non-WT genotype was set to 5% of the population size, the sampling parameters were set to $n_{s}=100$ and Δt=10, T=100 generation were used for inference, and the regularization parameter, γ, was set to one.

## Supplementary Material

msac199_Supplementary_Data

## Data Availability

Simulation data and a MATLAB (version R2017a) implementation of MPL used for reproducing results in the paper are freely available at https://github.com/mssohail/epistasis-inference.
