# Supplementary material for: Inferring Epistasis from Genetic Time-series Data
Source: Mol Biol Evol. 2022 Sep 21;39(10):msac199. doi: 10.1093/molbev/msac199 (PMC9558069; doi:10.1093/molbev/msac199)
Supplement: msac199_Supplementary_Data [file msac199_supplementary_data.zip › Supplementary_Material_MBE-22-0423.pdf]

SUPPLEMENTARY INFORMATION

Inferring epistasis from genetic time-series data

Muhammad Saqib Sohail[1], Raymond H. Y. Louie[2], Zhenchen Hong[3], John P. Barton[3,4,*], and Matthew R. McKay[1,5,6,7,*]

[1]Department of Electronic and Computer Engineering, Hong Kong University of Science and Technology, Hong Kong SAR, China
[2]The Kirby Institute, University of New South Wales, Sydney, New South Wales, Australia
[3]Department of Physics and Astronomy, University of California, Riverside, CA, USA
[4]Department of Computational and Systems Biology, University of Pittsburgh School of Medicine, Pittsburgh, PA, USA
[5]Department of Chemical and Biological Engineering, Hong Kong University of Science and Technology, Hong Kong SAR, China
[6]Department of Electrical and Electronic Engineering, University of Melbourne, Melbourne, Victoria, Australia
[7]Department of Microbiology and Immunology, University of Melbourne, at The Peter Doherty Institute for Infection and Immunity, Melbourne, Victoria, Australia
[*]Corresponding authors: matthew.mckay@unimelb.edu.au and jpbarton@pitt.edu

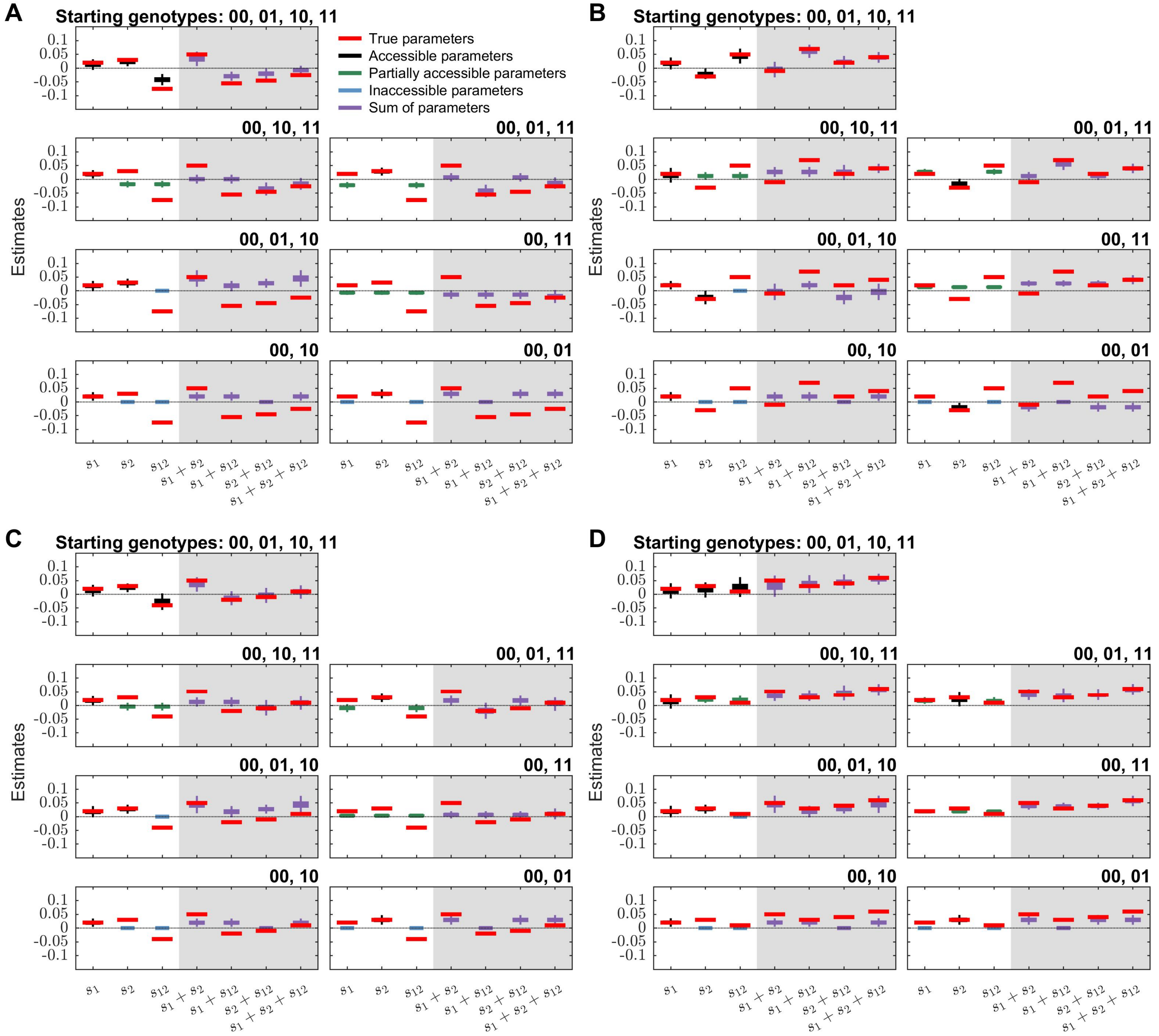

Figure S1 MPL can accurately estimate individual selection coefficients and pairwise epistasis terms and/or their sums depending on the variation present in the population. Results are for a two-locus system with (A) negative sign epistasis ($s_1 = 0.02, s_2 = 0.03, s_{12} = -0.075$), (B) positive sign epistasis ($s_1 = 0.02, s_2 = -0.03, s_{12} = 0.05$), (C) negative epistasis ($s_1 = 0.02, s_2 = 0.03, s_{12} = -0.04$), (D) positive epistasis ($s_1 = 0.02, s_2 = 0.03, s_{12} = 0.01$). All results were computed over 1000 Monte Carlo runs. The boxplots of inferred selection coefficients and epistasis terms are shown on white background in each panel, while those of their sums are shown on grey background. The red lines indicate the true values of the respective terms. The boxplots show the standard data summary (minimum, first quartile, median, third quartile, maximum). In order to control genetic diversity, both the per locus mutation probability and the per locus recombination probability were set to zero. The population size N was set to 1000, the initial population contained the genotypes indicated above each panel, and the frequency of each non-WT genotype in the initial population was set to 10% of the population size. The sampling parameters were set to $n_s = 100$ and $\Delta t = 10$, with $T = 150$ generation used for inference.

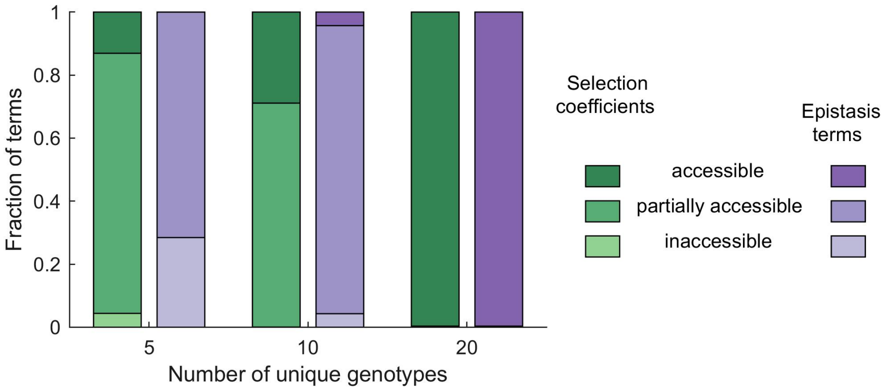

Figure S2 The average fraction of accessible selection coefficients and pairwise epistasis terms increases with increasing genetic diversity (controlled by changing the number of unique genotypes in initial population to either five, ten or twenty). The selection coefficients and pairwise epistasis terms of a five-locus system were classified into three categories based on the reduced row-Echelon form (see Materials and Methods) of the integrated covariance matrix in (21). Results are for the five-locus system (i.e., five selection coefficients and ten pairwise epistasis terms) simulated in Figure 4.

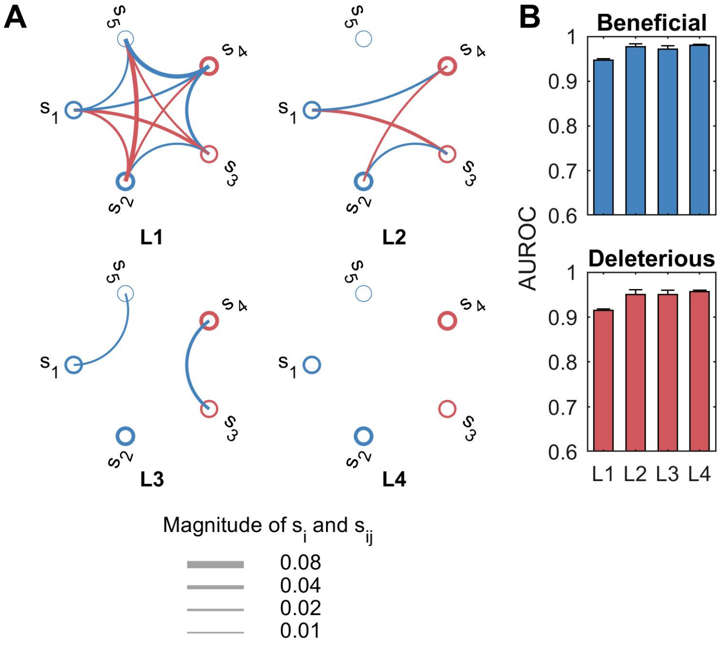

Figure S3 MPL performs well on dense as well as sparse fitness landscapes. (A) shows true (model) fitness landscapes with varying density of pairwise epistasis terms, while (B) shows the corresponding mean AUROC of detecting accessible beneficial and deleterious selection coefficients. Results are for a five-locus system where $N = 1000$, the initial population contained twenty unique genotypes, with per locus mutation probability $\mu = 10^{-4}$ and per locus recombination probability $r = 10^{-4}$. The selection coefficients at loci are shown by circles and pairwise epistasis terms by chords between loci (*blue*: beneficial and *red*: deleterious). Error bars indicate the standard error of the mean.

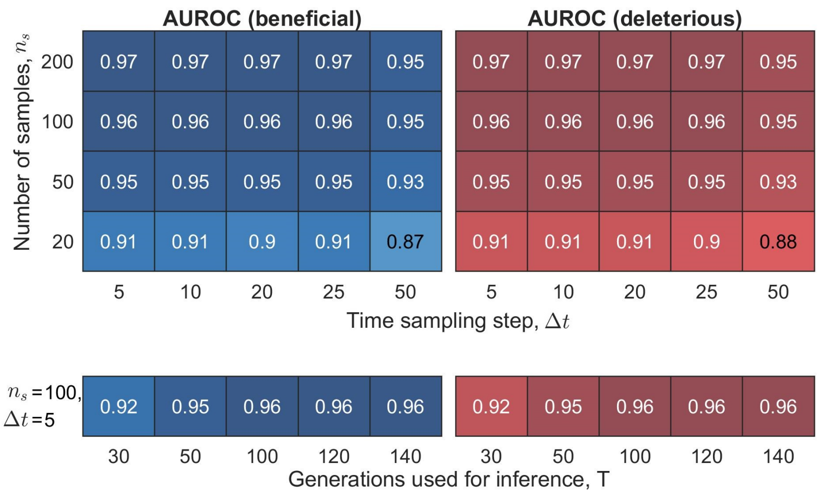

Figure S4 MPL is robust to variation in sampling parameters. The *left* and *right* panels show the mean AUROC performance of detecting accessible beneficial and deleterious epistasis terms respectively. The top panels show mean AUROC performance for a range of values of number of samples, n_s, and time sampling step, Δt, with a fixed value of number of generations used for inference, $T = 100$, while the bottom panels show the performance for a range of values of T with $n_s = 100$ and $\Delta t = 5$. Results are for a five-locus system with the fitness landscape shown in Figure 4A. The population size N was set to 1000. The initial population contained twenty unique genotypes. Other simulation parameters included per locus mutation probability $\mu = 10^{-4}$ and per locus recombination probability $r = 10^{-4}$. All results were averaged over 1000 Monte Carlo runs.

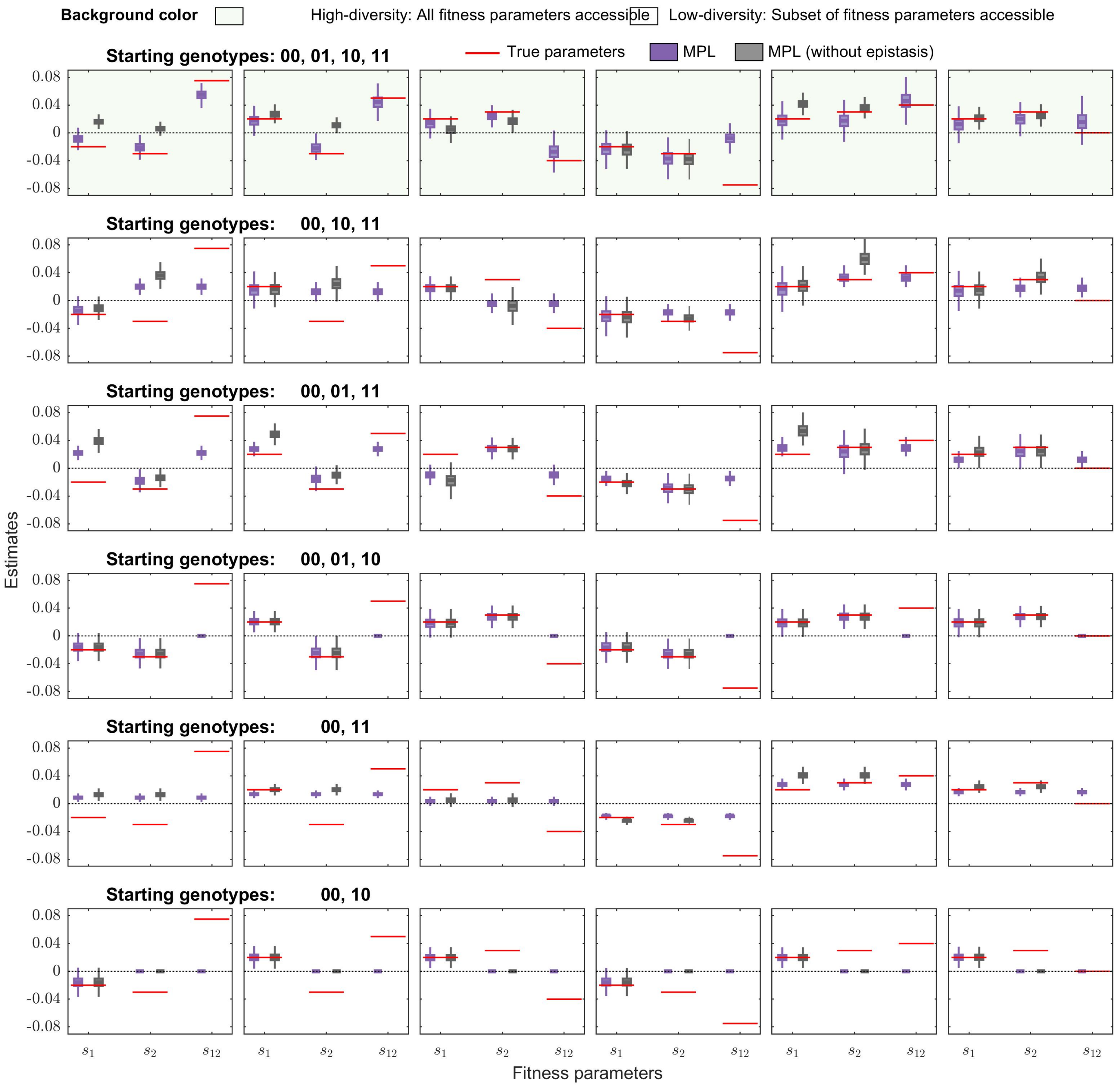

Figure S5 MPL has better performance than MPL (without epistasis) when epistasis exists in the fitness landscape and the data has enough genetic diversity to infer epistasis. Results are for a two-locus system with a range of different fitness landscapes and different levels of genetic diversity in the data. The boxplots show the standard data summary (first quartile, median, third quartile) with the whiskers showing 1.5 times the interquartile range. In order to control genetic diversity, both the per locus mutation probability and the per locus recombination probability were set to zero. The population size N was set to 1000. The panels in each row depict scenarios with different starting populations and the genotypes contained in the starting population are mentioned above each row, while the panels in each column depict scenarios with different fitness parameters. The frequency of each non-WT genotype in the initial population was set to 10% of the population size. The sampling parameters were set to $n_s = 100$, $\Delta t = 10$, and $T = 150$, where n_s is the number of samples, Δt is the time sampling step and T is the number of generations used for inference.

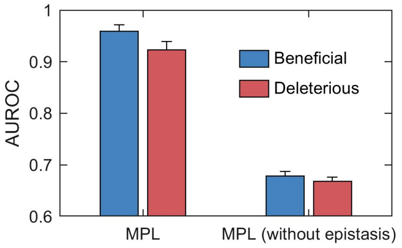

Figure S6 MPL outperforms the MPL (without epistasis) method in classification of beneficial and deleterious accessible selection coefficients even on data with low genetic diversity. The figure shows the mean AUROC performance of the two methods. Error bars indicate the standard error of the mean. Results are for a five-locus system with the fitness landscape shown in Figure 4A. The population size N was set to 1000 and the initial population contained five unique genotypes. The frequency of each non-WT genotype in the initial population was set to 5% of the population size. Both the per locus mutation probability μ and per locus recombination probability r set to zero. The sampling parameters were set to $n_s = 100$, $\Delta t = 10$, and $T = 100$, where n_s is the number of samples, Δt is the time sampling step and T is the number of generations used for inference. Results were computed over 1000 Monte Carlo runs.

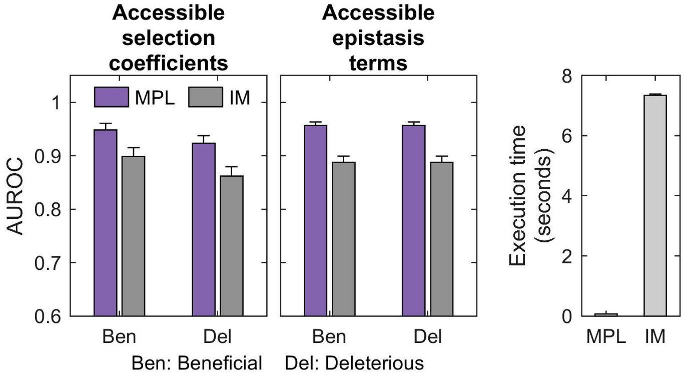

Figure S7 MPL has improved classification performance and improved computational efficiency compared with a state-of-the-art method of Illingworth *et al.* (2014) (IM). The *left* and *center* panels show the mean AUROC performance of detecting accessible selection coefficients and epistasis terms, respectively. The *right* panel shows the mean execution time of the two methods. Results are for a five-locus system with the fitness landscape shown in Figure 4A. The population size N was set to 1000 and the initial population contained 20 unique genotypes. Other simulation parameters included per locus mutation probability $\mu = 10^{-4}$ and per locus recombination probability $r = 10^{-4}$. The sampling parameters were set to $n_s = 100$ and $\Delta t = 10$, with $T = 100$ generation used for inference. All results were averaged over 100 Monte Carlo runs.

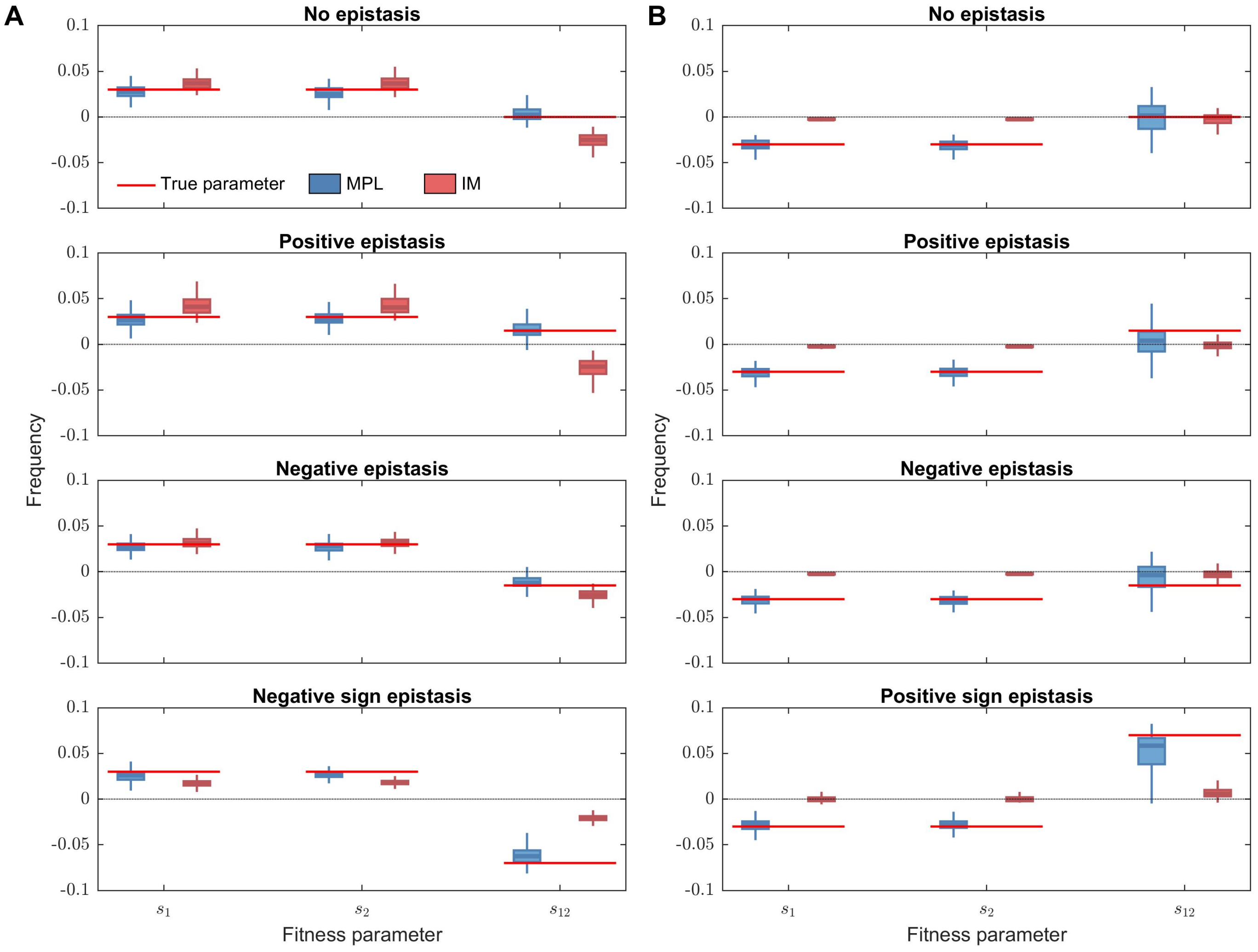

Figure S8 MPL outperforms a state-of-the-art method of Illingworth *et al.* (2014) (IM) in inferring fitness parameters. Results are for the same two-locus system with selection, mutation, and recombination as in Figure 1. (A) shows box-plots of inferred selection coefficients and pairwise epistasis terms by MPL and IM for various forms of epistasis when both selection coefficients are positive ($s_1 = s_2 = 0.03$), while (B) shows the same for the case when both selection coefficients are negative $s_1 = s_2 = -0.03$). The pairwise epistasis term s_{12} was set to $\{0, 0.015, -0.015, 0.07, -0.07\}$ to simulate the scenarios of no epistasis, positive epistasis, negative epistasis, positive sign epistasis, and negative sign epistasis respectively. Other simulation parameters included per locus mutation probability $\mu = 10^{-3}$, per locus recombination probability $r = 10^{-3}$, and population size $N = 1000$. The initial population consisted of only the WT genotype (00). The sampling parameters were set to $n_s = 100$, $\Delta t = 10$, and $T = 1000$, where n_s is the number of samples, Δt is the time sampling step and T is the number of generations used for inference. All simulation results were computed over 100 Monte Carlo runs. The solid red bars represent the true selection coefficients (s_1 and s_2) and epistasis term (s_{12}). The boxplots show the standard data summary (first quartile, median, third quartile) with the whiskers showing 1.5 times the interquartile range.

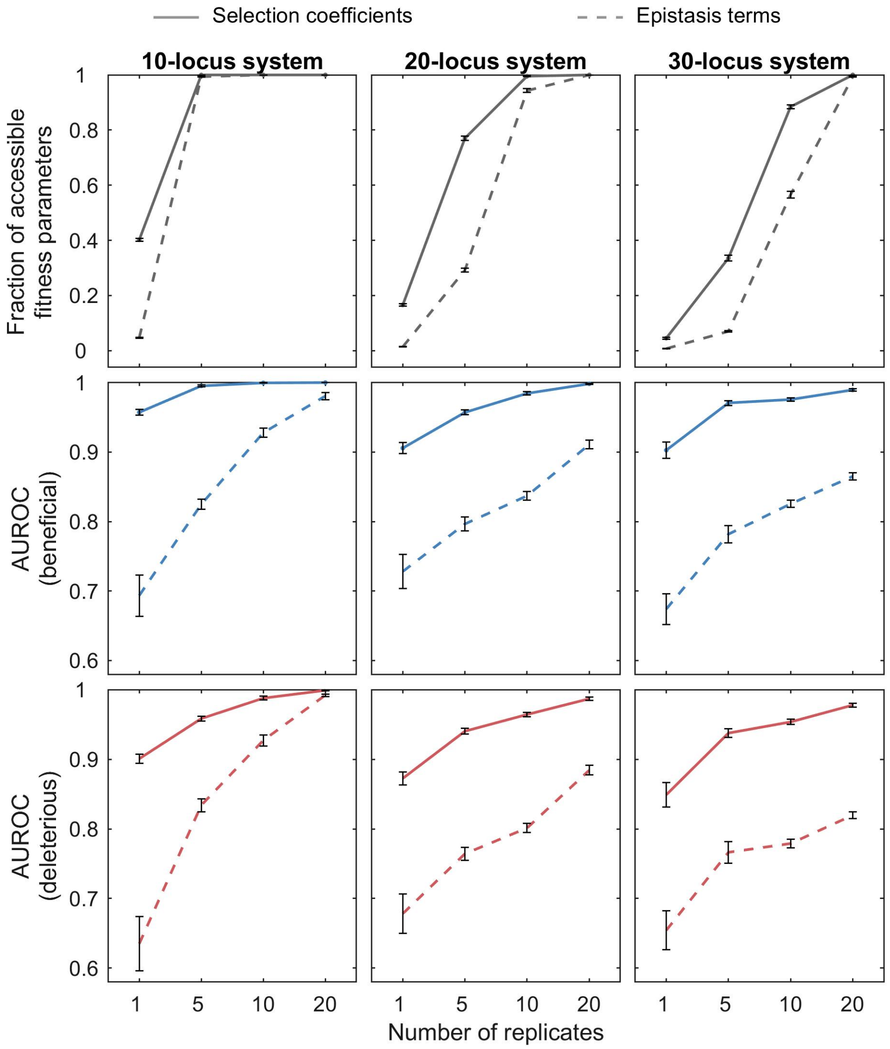

Figure S9 MPL can infer fitness parameters in large-sized systems, provided there is sufficient genetic diversity in data. The mean fraction of accessible fitness parameters (*top* panel) is presented, along with the mean AUROC performance of classifying accessible beneficial (*middle* panel) and deleterious (*bottom* panel) fitness parameters from the rest, computed over 1000 Monte Carlo runs. For each fitness landscape, the beneficial and the deleterious fitness parameters (selection coefficients and epistasis terms) were randomly drawn from uniform distributions over the ranges $[0.025, 0.075]$ and $[-0.025, -0.075]$, respectively. The fraction of beneficial and deleterious selection coefficients in the system was $\sim$ 35% each, while the rest were neutral. The fraction of non-zero epistasis terms was $\sim$ 15% in each simulation scenario, where approximately half of the non-zero epistasis terms were positive, while the other half were negative. The population size N was set to 1000 and the initial population contained 10 unique genotypes. Other simulation parameters included per locus mutation probability $\mu = 10^{-4}$ and per locus recombination probability $r = 10^{-4}$. The sampling parameters were set to $n_s = 100$ and $\Delta t = 10$, with $T = 100$ generation used for inference.

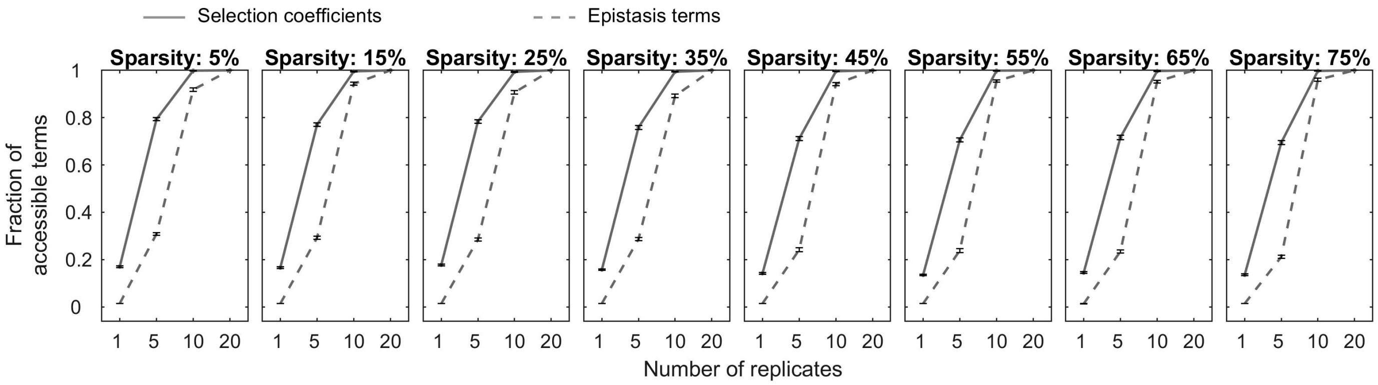

Figure S10 The fraction of accessible fitness parameters does not depend on the sparsity level of the fitness landscape. The mean fraction of accessible fitness parameters (selection coefficients and epistasis terms) is presented, for different sparsity levels, computed over 1000 Monte Carlo runs for a 20-locus system. For each fitness landscape, the beneficial and the deleterious fitness parameters were randomly drawn from uniform distributions over the ranges $[0.025, 0.075]$ and $[-0.025, -0.075]$, respectively. The fraction of beneficial and deleterious selection coefficients in the system was 35% each, while the rest were neutral. Sparsity here refers to the number of non-zero epistasis terms, approximately half of which were positive while the other half were negative. The population size N was set to 1000 and the initial population contained 10 unique genotypes. Other simulation parameters included per locus mutation probability $\mu = 10^{-4}$ and per locus recombination probability $r = 10^{-4}$. The sampling parameters were set to $n_s = 100$ and $\Delta t = 10$, with $T = 100$ generation used for inference.

′

Inference framework

Previously, we presented the MPL framework (Sohail *et al.* 2021) which allows derivation of closed-from expressions for the estimates of selection coefficients in a multi-locus system while accounting for effects of linkage, selection, mutation, recombination, and incomplete temporal sampling, under an additive fitness model. Here, we extend this framework to a fitness model with pairwise epistasis terms and derive the MPL estimate (21) of selection coefficient and epistasis terms. We also give the MPL estimate to combine multiple independent replicates (22) and to account for asymmetrical mutation probabilities under the epistatic fitness model. The key technical innovation in the present work is the modeling of the evolution of both the single and the double mutant allele frequencies which allows the estimation of the pairwise epistasis terms, as opposed to modeling the evolution of only the single allele frequencies in Sohail *et al.* (2021).

The MPL framework uses the path integral approach (Risken 1989) to efficiently compute the probability of an evolutionary path followed by the frequency vector (of all genotypes in the population) over time. While well known in physics (Risken 1989), the path integral approach is relatively less known in population genetics though a few exceptions exist (Mustonen and Lässig 2010; Schraiber 2014; Illingworth *et al.* 2011) where the path integral was utilized for purposes other than inference.

Path integral

We begin by describing the evolutionary model. Next, we give the expression for the genotype-level path integral representation as derived in Sohail *et al.* (2021). We then derive the allele level path integral for the case of epistatic fitness model and obtain the MPL estimate (21), followed by the MPL estimate to combine multiple replicates (22), and the MPL estimate for asymmetrical mutation probabilities.

Evolutionary model

The evolutionary model assumed here is the same as in Sohail *et al.* (2021) with the exception that here the fitness model also has pairwise epistasis terms. For completeness, we give the details of the model below.

We assume a WF model consisting of N individuals evolving under mutation, selection and recombination. Each individual is represented by a sequence of length L. The loci are assumed to be bi-allelic where the value of each locus is either 0 (wild-type (WT)) or 1 (mutant), thus resulting in $M = 2^L$ genotypes. For clarity, we use $i, j, \ldots$ to refer to locus indices and $a, b, \ldots$ to refer to genotype indices. The index is shown as a subscript when representing only one of the locus or genotype indices. However, when both indices need to be shown simultaneously, the locus index is shown as a subscript while the genotype index is shown as a superscript. Let $n_a(t)$ denote the number of individuals in the population that belong to genotype a at generation t such that $\sum_{a=1}^{M} n_a(t) = N$. At generation t, denote $\mathbf{Z}(t) = (Z_1(t), \ldots, Z_M(t))$ as the random genotype frequency vector, and $\mathbf{z}(t) = (z_1(t), \ldots, z_M(t))$ as an observed realization of this random vector with $z_a(t) = \frac{n_a(t)}{N}$.

Let r be the probability of recombination per locus per generation. The frequency of genotype a at generation t after recombination is given by

$$y_a(t) = (1-r)^{L-1} z_a(t) + \left(1 - (1-r)^{L-1}\right) \psi_a(\mathbf{z}(t)) \tag{S1}$$

where $(1-r)^{L-1} z_a(t)$ represents the fraction of genotype a not undergoing recombination, $\left(1 - (1-r)^{L-1}\right) \psi_a(\mathbf{z}(t))$ the fraction of genotype a arising as a result of recombination, and the factor $L-1$ arises as there are $L-1$ possible recombination breakpoints. The quantity $\psi_a(\mathbf{z}(t))$ is the probability that a recombination event results in an individual of genotype a and is a function of the composition of the population at generation t. We represent this quantity as

$$\psi_a(\mathbf{z}(t)) = \sum_{c=1}^{M} \sum_{d=1}^{M} R_{a,cd} z_c(t) z_d(t) \tag{S2}$$

where $R_{a,cd}$ is the probability that genotypes c and d recombine to form genotype a and is a function of the number of breakpoints and the particular genotypes a, c and d. We describe this in detail later in the document, when we calculate the recombination term in (S40) and (S46).

Under the WF dynamics, the probability of observing genotype frequencies $\mathbf{z}(t+1)$ at generation $t+1$, given genotype frequencies of $\mathbf{z}(t)$ at generation t, is given by (Ewens 2012)

$$P\left(\mathbf{z}(t+1)\middle|\mathbf{z}(t)\right) = N!\prod_{a=1}^{M}\frac{\left(p_a(\mathbf{z}(t))\right)^{Nz_a(t+1)}}{(Nz_a(t+1))!}. \tag{S3}$$

Here $p_a(\mathbf{z}(t))$ is given as

$$\begin{aligned} p_a(\mathbf{z}(t)) &:= \mathrm{E}\left[Z_a(t+1)\middle|\mathbf{Z}(t)=\mathbf{z}(t)\right] \\ &= \frac{y_a(t)f_a + \sum_{b=1,b\neq a}^{M}\left(\mu_{ba}y_b(t)f_b - \mu_{ab}y_a(t)f_a\right)}{\sum_{b=1}^{M}y_b(t)f_b}, \end{aligned} \tag{S4}$$

where μ_{ba} is the probability of genotype b mutating to genotype a, and f_a is the Wrightian fitness of the ath genotype. In contrast to the fitness model considered in Sohail *et al.* (2021), here we consider a fitness model that accounts for epistasis arising due to pairwise interactions between loci. The total fitness of a genotype is thus given by the sum of the independent effects of the individual loci and the pairwise interactions between all loci, i.e.,

$$\begin{aligned} f_a &= 1 + h_a \\ &= 1 + \sum_{i=1}^{L}s_i g_i^a + \sum_{i=1}^{L}\sum_{j=i+1}^{L}s_{ij}g_i^a g_j^a \end{aligned} \tag{S5}$$

where h_a, s_i, s_{ij} denote the time-invariant genotype selection coefficient, allele selection coefficient and epistasis terms respectively, and g_i^a represents the allele (either 0 or 1) at the ith locus of the ath genotype. We can compactly denote the selection coefficients and epistasis terms in a single vector as

$$\mathbf{s} = \left(s_1, \cdots, s_L, s_{12}, \cdots, s_{(L-1)L}\right) \tag{S6}$$

where the first L elements are the selection coefficients while the last $L(L-1)/2$ elements are pairwise epistasis terms. Similar to the notation adopted in the main text, we differentiate between non-italic and italic scalar notation to facilitate sequential indexing throughout the supplementary text. Thus we write

$$\mathbf{s} = \left(\mathrm{s}_1, \cdots, \mathrm{s}_L, \mathrm{s}_{L+1}, \cdots, \mathrm{s}_R\right) \tag{S7}$$

where $R = L(L+1)/2$ and we have $\mathrm{s}_e = s_i$ for $e \in \{1, \ldots, L\}$, and $\mathrm{s}_e = s_{ij}$ for $e \in \{L+1, \ldots, R\}$, with obvious association between indices e and (i, j).

For simplicity of exposition, we assume that the forward and backward mutation probabilities are equal, thus $\mu_{ba} = \mu_{ab} = \mu^{d_{ab}}$, where μ is the per locus mutation probability and d_{ab} the Hamming distance between genotypes a and b. Unequal forward and backward mutation probabilities can also be adapted in our model as shown further ahead in this supplement. We may then write

$$p_a(\mathbf{z}(t)) = \frac{(1+h_a)\,y_a(t) + \sum\limits_{b=1}^{M}\mu^{d_{ab}}\left((1+h_b)\,y_b(t) - (1+h_a)\,y_a(t)\right)}{\sum\limits_{b=1}^{M}(1+h_b)\,y_b(t)}. \tag{S8}$$

The probability that the genotype frequency vector evolving over T generations, where samples are collected during non-successive generations $t_k \in \{0, 1, \cdots, K\}$, follows a particular evolutionary path $(\mathbf{z}(t_1), \mathbf{z}(t_2), \cdots, \mathbf{z}(t_K))$ conditioned on the initial state $\mathbf{z}(t_0)$ is given by

$$P\left((\mathbf{z}(t_k))_{k=1}^{K}\,|\mathbf{z}(t_0)\right) = \prod_{k=0}^{K-1}P\left(\mathbf{z}(t_{k+1})|\mathbf{z}(t_k)\right). \tag{S9}$$

In principle, we can use the above expression to infer evolutionary parameters. However, the expression above is unyielding for the purpose of parameter inference due to the large dimensionality of the genotype space, which grows exponentially with sequence length, as well as intractability of the fractional form of the right hand side of (S9).

To simplify the problem, we use a path integral to approximate the probability in (S9). The first step of the approach consists of approximating the WF process by a diffusion process, as commonly done in population genetics (Kimura 1964; Ewens 2012; Tataru *et al.* 2015; He *et al.* 2017; Tataru *et al.* 2017). Specifically, assume the population is large and that

$$s_e = \frac{\bar{s}_e}{N} + O\left(\frac{1}{N^2}\right), \quad \mu = \frac{\bar{\mu}}{N} + O\left(\frac{1}{N^2}\right) \quad r = \frac{\bar{r}}{N} + O\left(\frac{1}{N^2}\right), \tag{S10}$$

and consequently

$$h_a = \frac{\bar{h}_a}{N} + O\left(\frac{1}{N^2}\right), \tag{S11}$$

where $\bar{s}_e$, $\bar{\mu}$, $\bar{r}$, and $\bar{h}_a$ are constants that are independent of N. Under this scaling, we have

$$y_a(t) = z_a(t) - r(L-1)\big(z_a(t) - \psi_a(\mathbf{z}(t)\big) + O\left(\frac{1}{N^2}\right) \tag{S12}$$

where L is also assumed to be constant with regards to N.

Genotype-level path integral

In Sohail *et al.* (2021), we derived the genotype-level path integral to approximate the probability of observing a trajectory of genotype frequencies $(\mathbf{z}(t_1), \mathbf{z}(t_2), \cdots, \mathbf{z}(t_K))$. Since this analysis applies equally to the current work, we give a brief summary here. We approximated the transition probability of the WF evolutionary process, using standard diffusion theory (Ewens 2012), by the transition probability density of a diffusion process, i.e.,

$$\check{\mathbf{Z}}(\tau) = \big(\check{Z}_1(\tau), \ldots, \check{Z}_M(\tau)\big) := \mathbf{Z}(\lfloor N\tau \rfloor), \quad \tau \geq 0 \tag{S13}$$

taken in the limit $N \to \infty$. Here $\lfloor \cdot \rfloor$ denotes the floor function and τ is a continuous time variable with units of N generations, with one generation in discrete time (i.e., from t to $t+1$) thus taking

$$\delta\tau = \frac{1}{N} \tag{S14}$$

continuous time units. The genotype-level diffusion process was found to be characterized by the drift vector $\bar{d}(\check{\mathbf{z}}(\tau))$ with ath entry

$$\bar{d}_a(\check{\mathbf{z}}(\tau)) = \check{z}_a(\tau)\left(\bar{h}_a - \sum_{b=1}^{M} \bar{h}_b \check{z}_b(\tau)\right) + \bar{\mu}\left(\sum_{b=1, d_{ab}=1}^{M} \check{z}_b(\tau) - \sum_{b=1, d_{ab}=1}^{M} \check{z}_a(\tau)\right) - \bar{r}(L-1)\big(\check{z}_a(\tau) - \psi_a\left(\check{\mathbf{z}}(\tau)\right)\big), \tag{S15}$$

and diffusion matrix $\bar{C}(\check{\mathbf{z}}(\tau))$ with (a,b)th entry

$$\bar{C}_{ab}(\check{\mathbf{z}}(\tau)) = \frac{1}{2}\begin{cases} \check{z}_a(\tau)(1 - \check{z}_a(\tau)) & a = b \\ -\check{z}_a(\tau)\check{z}_b(\tau) & a \neq b. \end{cases} \tag{S16}$$

The time evolution of the transition probability density of the diffusion process $\check{\mathbf{Z}}(\tau)$ is described by the Kolmogorov forward equation (also known as the Fokker-Planck equation). Discretizing the transition probability density of the diffusion over small $\delta\tau\Delta t$ (equivalently large $\frac{N}{\Delta t}$), we approximated the probability of observing a trajectory of genotype frequencies $(\mathbf{z}(t_1), \mathbf{z}(t_2), \cdots, \mathbf{z}(t_K))$ conditioned on $\mathbf{z}(t_0)$ as

$$\begin{aligned} P\left((\mathbf{z}(t_k))_{k=1}^{K} \,|\mathbf{z}(t_0)\right) &= \prod_{k=0}^{K-1} P(\mathbf{z}(t_{k+1})|\mathbf{z}(t_k)) \\ &\approx \prod_{k=0}^{K-1}\left[\frac{1}{\sqrt{\det C(\mathbf{z}(t_k))}}\left(\frac{N}{2\pi\Delta t_k}\right)^{M/2}\prod_{a=1}^{M} \mathrm{d}z_a(t_{k+1})\right]\exp\left(-\frac{N}{2}S\left((\mathbf{z}(t_k))_{k=0}^{K}\right)\right) \end{aligned} \tag{S17}$$

with

$$S\left((\mathbf{z}(t_k))_{k=0}^{K}\right) = \sum_{k=0}^{K-1}\frac{1}{\Delta t_k}\sum_{a=1}^{M}\sum_{b=1}^{M}\left[z_a(t_{k+1}) - z_a(t_k) - d_a(\mathbf{z}(t_k))\Delta t_k\right]\left(C^{-1}(\mathbf{z}(t_k))\right)_{ab}\left[z_b(t_{k+1}) - z_b(t_k) - d_b(\mathbf{z}(t_k))\Delta t_k\right],$$

where $\Delta t_k = t_{k+1} - t_k$ and we have defined $d_a(\mathbf{z}(t_k)) := \frac{\bar{d}_a(\mathbf{z}(t_k))}{N}$, $(C(\mathbf{z}(t_k)))_{ab} := 2\left(\bar{C}(\mathbf{z}(t_k))\right)_{ab}$. This is the path integral representation of the genotype dynamics (see Supplement of Sohail *et al.* (2021) for a detailed derivation).

Allele-level path integral

In Sohail *et al.* (2021), we modeled the evolution of the single mutant frequencies by applying linear combinations to the genotype frequencies described by (S17), while assuming the double mutant frequencies were known. The single and double mutant frequencies relate to the genotype frequencies via

$$x_i(t) = \sum_{a=1}^{M} g_i^a z_a(t), \qquad x_{ij}(t) = \sum_{a=1}^{M} g_i^a g_j^a z_a(t), \tag{S18}$$

where $x_i(t)$, and $x_{ij}(t)$, are the single and the double mutant frequencies at locus i and locus-pair (i,j) respectively at generation t. Here, in contrast, we model the evolution of both the single and double mutant frequencies which additionally requires the knowledge of the triple and quadruple mutant frequencies. These are related to the genotype frequencies via

$$x_{ijk}(t) = \sum_{a=1}^{M} g_i^a g_j^a g_k^a z_a(t), \quad x_{ijkl}(t) = \sum_{a=1}^{M} g_i^a g_j^a g_k^a g_l^a z_a(t), \tag{S19}$$

where $x_{ijk}(t)$ and $x_{ijkl}(t)$ are the triple and the quadruple mutant frequencies at locus-triplet (i,j,k) and locus-quartet (i,j,k,l) respectively at generation t. We concatenate the single and double mutant allele frequencies in a R length vector, where $R = L(L+1)/2$, as

$$\mathbf{x}(t) = \Big(x_1(t), \cdots, x_L(t), x_{12}(t), x_{13}(t), \cdots, x_{(L-1)L}(t)\Big). \tag{S20}$$

Similar to the notation in the main text, we write

$$\mathbf{x}(t) = \Big(\mathrm{x}_1(t), \cdots, \mathrm{x}_L(t), \mathrm{x}_{L+1}(t), \cdots, \mathrm{x}_R(t)\Big) \tag{S21}$$

to facilitate sequential indexing for notation convenience. Note that we differentiate between non-italic and italic scalar notation, as described in (S6) and (S7). Clearly, from (S20) and (S21), we have $\mathrm{x}_e(t) = x_i(t)$ for $e \leq L$, and $\mathrm{x}_e(t) = x_{ij}(t)$ for $L < e \leq R$.

To simplify the presentation, we also define U as an $M \times R$ mapping matrix where the ath row of U, i.e., $\mathbf{u}_a = (\mathrm{u}_1^a, \cdots, \mathrm{u}_L^a, \mathrm{u}_{L+1}^a, \cdots, \mathrm{u}_R^a)$, is given by

$$\mathbf{u}_a = \Big(g_1^a, \cdots, g_L^a, g_1^a g_2^a, \cdots, g_1^a g_L^a, g_2^a g_3^a, \cdots, g_2^a g_L^a, \cdots, g_{L-1}^a g_L^a\Big). \tag{S22}$$

Note that g_i^a refers to the allele at the ith locus while $g_i^a g_j^a$ refers to the pair of alleles at locus-pair (i,j) in genotype a.

Next, we define a random vector comprising of the single and double mutant allele frequencies, i.e., $\mathbf{X}(t) = \Big(X_1(t), \ldots, X_L(t), X_{12}(t), \ldots, X_{(L-1)L}(t)\Big)$, which from (S18) is related to the random genotype frequency vector by

$$\mathrm{X}_e(t) = \sum_{a=1}^{M} \mathrm{u}_e^a Z_a(t) \tag{S23}$$

where u_e^a denotes the eth entry of $\mathbf{u}_a$.

Thus, $\mathbf{x}(t)$ is a realization of the random vector $\mathbf{X}(t)$. Similarly, the allele-level continuous process can be shown to be related to the genotype-level continuous time process (S13) using the transformation above, and is given as

$$\check{\mathbf{X}}(\tau) = \Big(\check{X}_1(\tau), \ldots, \check{X}_L(\tau), \check{X}_{12}(\tau)\check{X}_{13}(\tau), \ldots, \check{X}_{(L-1)L}(\tau)\Big) := \mathbf{X}(\lfloor N\tau \rfloor), \quad \tau \geq 0 \tag{S24}$$

taken as $N \to \infty$.

The time evolution of the transition probability density, ϕ, of the allele-level diffusion, is governed by the Kolmogorov forward equation

$$\frac{\partial \phi}{\partial \tau} = \left[-\sum_{e=1}^{R} \frac{\partial}{\partial \check{\mathrm{x}}_e} \bar{\mathrm{d}}_e(\check{\mathbf{x}}(\tau)) + \sum_{e=1}^{R} \sum_{f=1}^{R} \frac{\partial}{\partial \check{\mathrm{x}}_e} \frac{\partial}{\partial \check{\mathrm{x}}_f} \bar{\mathrm{C}}_{ef}(\check{\mathbf{x}}(\tau)) \right] \phi, \tag{S25}$$

where $\bar{C}(\check{\mathbf{x}}(\tau))$ and $\bar{\mathbf{d}}(\check{\mathbf{x}}(\tau))$ are the diffusion matrix and the drift vector associated with the allele-level diffusion process that describes the conditional change in the single and double mutant frequencies.

The diffusion matrix of the allele-level diffusion process is of size $R \times R$ and can be partitioned into four sub-matrices, i.e., the upper left $L \times L$ matrix, the upper right $L \times \frac{L(L-1)}{2}$ matrix, lower left $\frac{L(L-1)}{2} \times L$ matrix and the lower right $\frac{L(L-1)}{2} \times \frac{L(L-1)}{2}$ matrix. The definition and interpretation of these matrices is given below. Recalling (S22), we note that the eth element of $\mathbf{u}_a$ refers to the allele at locus i for $1 \leq e \leq L$, and to the alleles at locus-pair (i, j) for $L < e \leq R$, i.e.,

$$u_e^a = \begin{cases} g_i^a & 1 \leq e \leq L \\ g_i^a g_j^a & L < e \leq R. \end{cases} \tag{S26}$$

The elements of the upper left sub-matrix of the diffusion matrix $\bar{C}(\check{\mathbf{x}}(\tau))$, i.e., $1 \leq e \leq L$ and $1 \leq f \leq L$, are given as

$$\begin{aligned} \bar{C}_{ef}(\check{\mathbf{x}}(\tau)) &:= \sum_{a=1}^{M}\sum_{b=1}^{M} u_e^a u_f^b \bar{C}_{ab}(\check{\mathbf{z}}(\tau)) \\ &= \frac{1}{2}\sum_{a=1}^{M} g_i^a g_j^a \frac{\check{z}_a(\tau)(1-\check{z}_a(\tau))}{N} - \frac{1}{2}\sum_{a=1}^{M}\sum_{b=1, b\neq a}^{M} g_i^a g_j^b \frac{\check{z}_a(\tau)\check{z}_b(\tau)}{N} + O\left(\frac{1}{N^2}\right) \\ &= \frac{1}{2}\frac{\check{x}_{ij}(\tau) - \check{x}_i(\tau)\check{x}_j(\tau)}{N} + O\left(\frac{1}{N^2}\right), \end{aligned} \tag{S27}$$

which measure the scaled joint variability between the number of mutants at loci i and j. We note here that the upper left sub-matrix here is the same as the diffusion matrix in Sohail *et al.* (2021) where only the evolution of the single mutant allele frequency was modeled.

Following similar steps, it can be shown that the entries of the upper right sub-matrix of the diffusion matrix $\bar{C}(\check{\mathbf{x}}(\tau))$, i.e., for $1 \leq e \leq L$ and $L < f \leq R$, are given as

$$\begin{aligned} \bar{C}_{ef}(\check{\mathbf{x}}(\tau)) &:= \sum_{a=1}^{M}\sum_{b=1}^{M} u_e^a u_f^b \bar{C}_{ab}(\check{\mathbf{z}}(\tau)) \\ &= \sum_{a=1}^{M}\sum_{b=1}^{M} g_i^a g_j^b g_k^b \bar{C}_{ab}(\check{\mathbf{z}}(\tau)) \\ &= \frac{1}{2}\frac{\check{x}_{ijk}(\tau) - \check{x}_i(\tau)\check{x}_{jk}(\tau)}{N} + O\left(\frac{1}{N^2}\right), \end{aligned} \tag{S28}$$

which measures the scaled joint variability between the number of mutants at locus i and double-mutants at loci j and k. Here $\check{x}_{ijk}(\tau)$ denotes the triple mutant frequency obtained by the transformations (S18) and (S19) with

$$\check{x}_{ijk}(\tau) := x_{ijk}(\lfloor N\tau \rfloor), \quad \tau \geq 0. \tag{S29}$$

The $\frac{L(L-1)}{2} \times L$ lower left sub-matrix is just the transpose of the $L \times \frac{L(L-1)}{2}$ upper right matrix. Similarly, the entries of the bottom right sub-matrix of the diffusion matrix $\bar{C}(\check{\mathbf{x}}(\tau))$, i.e., $L < e \leq R$ and $L < e \leq R$, are given as

$$\begin{aligned} \bar{C}_{ef}(\check{\mathbf{x}}(\tau)) &:= \sum_{a=1}^{M}\sum_{b=1}^{M} u_e^a u_f^b \bar{C}_{ab}(\check{\mathbf{z}}(\tau)) \\ &= \sum_{a=1}^{M}\sum_{b=1}^{M} g_i^a g_j^a g_k^b g_l^b \bar{C}_{ab}(\check{\mathbf{z}}(\tau)) \\ &= \frac{1}{2}\frac{\check{x}_{ijkl}(\tau) - \check{x}_{ij}(\tau)\check{x}_{kl}(\tau)}{N} + O\left(\frac{1}{N^2}\right), \end{aligned} \tag{S30}$$

which measures the scaled joint variability between the number of double-mutants at loci i and j, and double-mutants at loci k and l, with $\check{x}_{ijkl}(\tau)$ denoting the quadruple mutant frequency with

$$\check{x}_{ijkl}(\tau) := x_{ijkl}(\lfloor N\tau \rfloor), \quad \tau \geq 0. \tag{S31}$$

Note that while the diffusion matrix also depends on the dynamics of the triple and quadruple mutant frequencies, we only explicitly show the dependence on the single and double mutant frequencies for simplicity of notation.

We can show that the allele-level drift vector is a linear transformation of the genotype drift vector $\bar{d}_a(\check{\mathbf{z}}(\tau))$ defined in (S15). Recalling (S18), (S19), and noting that we can express h_a in (S5) as

$$h_a = \sum_{e=1}^{R} \mathrm{u}_e^a \mathrm{s}_e, \tag{S32}$$

the eth element of the allele-level drift vector is defined as

$$\begin{aligned}
\bar{\mathrm{d}}_e(\check{\mathbf{x}}(\tau)) &:= \sum_{a=1}^{M} \mathrm{u}_e^a \bar{d}_a(\check{\mathbf{z}}(\tau)) \\
&= \sum_{a=1}^{M} \mathrm{u}_e^a \left(\check{z}_a(\tau) \left(\bar{h}_a - \sum_{b=1}^{M} \bar{h}_b \check{z}_b(\tau) \right) + \bar{\mu} \left(\sum_{b=1, d_{ab}=1}^{M} \check{z}_b(\tau) - \sum_{b=1, d_{ab}=1}^{M} \check{z}_a(\tau) \right) - \bar{r}(L-1)\big(\check{z}_a(\tau) - \psi_a(\check{\mathbf{z}}(\tau))\big) \right) \\
&= \sum_{a=1}^{M} \mathrm{u}_e^a \Bigg(\check{z}_a(\tau)\big(1 - \check{z}_a(\tau)\big)\bar{h}_a - \check{z}_a(\tau) \sum_{b=1, b\neq a}^{M} \bar{h}_b \check{z}_b(\tau) + \bar{\mu} \left(\sum_{b=1, d_{ab}=1}^{M} \check{z}_b(\tau) - \sum_{b=1, d_{ab}=1}^{M} \check{z}_a(\tau) \right) - \bar{r}(L-1)\big(\check{z}_a(\tau) - \psi_a(\check{\mathbf{z}}(\tau)) \\
&= \check{\mathrm{x}}_e(\tau)\,(1 - \check{\mathrm{x}}_e(\tau))\,\bar{\mathrm{s}}_e + \sum_{f\neq e} \bar{\mathrm{C}}_{ef}(\check{\mathbf{x}}(\tau))\bar{\mathrm{s}}_f + \bar{\mu}\mathrm{v}_e(\check{\mathbf{x}}(\tau)) + \bar{r}\,\eta_e(\check{\mathbf{x}}(\tau)).
\end{aligned} \tag{S33}$$

The transformation of the third and the fourth terms on the right hand side of (S33), referred to here as the mutation term $\mathrm{v}_e(\check{\mathbf{x}}(\tau))$ and the recombination term $\eta_e(\check{\mathbf{x}}(\tau))$ respectively, is non-trivial and requires some algebraic manipulation which we detail below. We note here that the first L entries of $\bar{\mathrm{d}}_e(\check{\mathbf{x}}(\tau))$ constitute the drift vector of Sohail *et al.* (2021). While the transformation of the first L entries mutation and recombination terms were derived in the Supplementary Information of Sohail *et al.* (2021), we reproduce these here as they aid in understanding the notation and subsequent derivation of remaining entries $L < e \leq R$ of the mutation and recombination terms.

Calculating the mutation term: Here, we show the computations involved with the mutation term in going from the second last line of (S33) to the last line of (S33). First consider the case $1 \leq e \leq L$, for which

$$\begin{aligned}
\mathrm{v}_e(\check{\mathbf{x}}(\tau)) &= \sum_{a=1}^{M} \mathrm{u}_e^a \left(\sum_{b=1, d_{ab}=1}^{M} \check{z}_b(\tau) - \sum_{b=1, d_{ab}=1}^{M} \check{z}_a(\tau) \right) \\
&= \sum_{a=1}^{M} g_i^a \left(\sum_{b=1, d_{ab}=1}^{M} \check{z}_b(\tau) - \sum_{b=1, d_{ab}=1}^{M} \check{z}_a(\tau) \right) \\
&= \sum_{a=1}^{M} \left(\sum_{b=1, d_{ab}=1}^{M} g_i^a(1 - g_i^b)\check{z}_b(\tau) + \sum_{b=1, d_{ab}=1}^{M} g_i^a g_i^b \check{z}_b(\tau) - \sum_{b=1, d_{ab}=1}^{M} g_i^a(1 - g_i^b)\check{z}_a(\tau) - \sum_{b=1, d_{ab}=1}^{M} g_i^a g_i^b \check{z}_a(\tau) \right) \\
&= \sum_{a=1}^{M} \sum_{b=1, d_{ab}=1}^{M} g_i^a(1 - g_i^b)\,(\check{z}_b(\tau) - \check{z}_a(\tau)) + \sum_{a=1}^{M} \sum_{b=1, d_{ab}=1}^{M} g_i^a g_i^b\,(\check{z}_b(\tau) - \check{z}_a(\tau))\,.
\end{aligned} \tag{S34}$$

Where the second last line above follows from noting that the first summation on the right side of the third last line can be decomposed into two parts. The first where genotypes a and b differ only at locus i, and hence mutation of genotype b to genotype a changes the mutant allele frequency at locus i. The second is where the two genotypes differ from each other at a locus other than i and hence a mutation from genotype b to a does not effect the mutant allele frequency at locus i. Similarly, the second summation in the third last line can also be split into two parts. Now, note that

$$\sum_{a=1}^{M} \sum_{b=1, d_{ab}=1}^{M} g_i^a g_i^b\,(\check{z}_b(\tau) - \check{z}_a(\tau)) = 0,$$

as this quantity represents the mutation of those genotypes b to genotype a where both a and b have the mutant allele at locus i, while

$$\sum_{a=1}^{M} \sum_{b=1, d_{ab}=1}^{M} g_i^a(1 - g_i^b)\,(\check{z}_b(\tau) - \check{z}_a(\tau)) = 1 - 2\check{x}_i(\tau),$$

which represents the flow of mutational probabilities between the WT and the mutation allele, i.e., the mutational flux. Substituting the above two equations back in (S34) yields

$$\mathrm{v}_e(\check{\mathbf{x}}(\tau)) = 1 - 2\check{x}_i(\tau) \qquad \text{for} \quad 1 \leq e \leq L. \tag{S35}$$

Now consider the case when $L < e \leq R$ where we have

$$\begin{aligned}
\mathrm{v}_e(\check{\mathbf{x}}(\tau)) &= \sum_{a=1}^{M} \mathrm{u}_e^a \left(\sum_{b=1, d_{ab}=1}^{M} \check{z}_b(\tau) - \sum_{b=1, d_{ab}=1}^{M} \check{z}_a(\tau) \right) \\
&= \sum_{a=1}^{M} g_i^a g_j^a \left(\sum_{b=1, d_{ab}=1}^{M} \check{z}_b(\tau) - \sum_{b=1, d_{ab}=1}^{M} \check{z}_a(\tau) \right) \\
&= \sum_{a=1}^{M} \sum_{b=1, d_{ab}=1}^{M} g_i^a g_j^a (1 - g_i^b)(1 - g_j^b) \left(\check{z}_b(\tau) - \check{z}_a(\tau) \right) + \sum_{a=1}^{M} \sum_{b=1, d_{ab}=1}^{M} g_i^a g_j^a (1 - g_i^b) g_j^b \left(\check{z}_b(\tau) - \check{z}_a(\tau) \right) \\
&\quad + \sum_{a=1}^{M} \sum_{b=1, d_{ab}=1}^{M} g_i^a g_j^a g_i^b (1 - g_j^b) \left(\check{z}_b(\tau) - \check{z}_a(\tau) \right) + \sum_{a=1}^{M} \sum_{b=1, d_{ab}=1}^{M} g_i^a g_j^a g_i^b g_j^b \left(\check{z}_b(\tau) - \check{z}_a(\tau) \right). \qquad \text{(S36)}
\end{aligned}$$

Here, the summations on the right side of the last line above represent the net mutational flow to genotypes that contain alleles $(1,1)$ at locus-pair (i,j), from those genotypes that have alleles $(0,0)$, $(0,1)$, $(1,0)$ and $(1,1)$ respectively at locus-pair (i,j). We note that

$$\begin{aligned}
\sum_{a=1}^{M} \sum_{b=1, d_{ab}=1}^{M} g_i^a g_j^a (1 - g_i^b)(1 - g_j^b) \left(\check{z}_b(\tau) - \check{z}_a(\tau) \right) &= 0 \\
\sum_{a=1}^{M} \sum_{b=1, d_{ab}=1}^{M} g_i^a g_j^a (1 - g_i^b) g_j^b \left(\check{z}_b(\tau) - \check{z}_a(\tau) \right) &= \left(\check{x}_{ij}^{01}(\tau) - \check{x}_{ij}^{11}(\tau) \right) \\
\sum_{a=1}^{M} \sum_{b=1, d_{ab}=1}^{M} g_i^a g_j^a g_i^b (1 - g_j^b) \left(\check{z}_b(\tau) - \check{z}_a(\tau) \right) &= \left(\check{x}_{ij}^{10}(\tau) - \check{x}_{ij}^{11}(\tau) \right) \\
\sum_{a=1}^{M} \sum_{b=1, d_{ab}=1}^{M} g_i^a g_j^a g_i^b g_j^b \left(\check{z}_b(\tau) - \check{z}_a(\tau) \right) &= 0
\end{aligned}$$

where we use the notation $\check{x}_{ij}^{10}(\tau)$ to refer to mutant with alleles $(1,0)$ and locus-pair (i,j). The first equation equals zero as the probability of more than one mutation in a sequence is negligibly small $O(\frac{1}{N^2})$ under the diffusion approximation. While the last equation equals zero as it represents the mutational flow of all those genotypes where both genotypes a and b contain the alleles $(1,1)$ at locus-pair (i,j). Substituting the above in (S36) we have

$$\begin{aligned}
\mathrm{v}_e(\check{\mathbf{x}}(\tau)) &= \check{x}_{ij}^{01}(\tau) + \check{x}_{ij}^{10}(\tau) - 2\check{x}_{ij}^{11}(\tau) \\
&= \check{x}_{ij}^{01}(\tau) + \check{x}_{ij}^{11}(\tau) + \check{x}_{ij}^{10}(\tau) + \check{x}_{ij}^{11}(\tau) - 4\check{x}_{ij}^{11}(\tau) \\
&= \check{x}_i^{1}(\tau) + \check{x}_j^{1}(\tau) - 4\check{x}_{ij}^{11}(\tau) \\
&= \check{x}_i(\tau) + \check{x}_j(\tau) - 4\check{x}_{ij}(\tau), \qquad \text{(S37)}
\end{aligned}$$

where we have dropped the superscripts in the last line. Thus, from (S35) and (S37) we have

$$\mathrm{v}_e(\check{\mathbf{x}}(\tau)) = \begin{cases} 1 - 2\check{x}_i(\tau) & 1 \leq e \leq L \\ \check{x}_i(\tau) + \check{x}_j(\tau) - 4\check{x}_{ij}(\tau) & L < e \leq R, \end{cases} \qquad \text{(S38)}$$

where i and j are the subscript indices corresponding to the eth element of $\check{\mathbf{x}}(\tau)$.

Calculating the recombination term: Next we show the computations involved with the recombination term in going from the second last line of (S33) to the last line of (S33). First consider the case $1 \le e \le L$, for which

$$\begin{aligned}
\eta_e(\check{\mathbf{x}}(\tau)) &= \sum_{a=1}^{M} \mathbf{u}_e^a \Big((L-1)\big(\check{z}_a(\tau) - \psi_a\big(\check{\mathbf{z}}(\tau)\big)\Big) \\
&= \sum_{a=1}^{M} g_i^a \Big((L-1)\big(\check{z}_a(\tau) - \psi_a\big(\check{\mathbf{z}}(\tau)\big)\Big) \\
&= (L-1)\check{x}_i(\tau) - (L-1) \sum_{a=1}^{M} g_i^a \psi_a\big(\check{\mathbf{z}}(\tau)\big) \\
&= (L-1)\check{x}_i(\tau) - (L-1) \sum_{a=1}^{M} g_i^a \sum_{c=1}^{M} \sum_{d=1}^{M} R_{a,cd} \check{z}_c(\tau) \check{z}_d(\tau),
\end{aligned} \tag{S39}$$

where the second line follows from (S22), and the last line follows by substituting the definition of $\psi_a\big(\check{\mathbf{z}}(\tau)\big)$ from (S2). To further simplify, let

$$\theta_i^{cd} := \sum_{a=1}^{M} g_i^a R_{a,cd} \tag{S40}$$

which is the probability that genotypes c and d recombine to form a genotype that has a mutation at locus i. For the bi-allelic model considered here, there are four possible scenarios for a recombination event: both genotypes c and d have allele 1 at their respective i-th locus, one of the genotypes has allele 1 while the other has allele 0, or both genotypes have allele 0 at the i-th locus. We partition the summation term on the right side of (S39) into these four recombination scenarios as follows

$$\begin{aligned}
\sum_{a=1}^{M} g_i^a \sum_{c=1}^{M} \sum_{d=1}^{M} R_{a,cd} \check{z}_c(\tau) \check{z}_d(\tau) &= \sum_{c=1}^{M} \sum_{d=1}^{M} \theta_i^{cd} \check{z}_c(\tau) \check{z}_d(\tau) \\
&= \sum_{c=1}^{M} \left(\sum_{d=1}^{M} g_i^c g_i^d \theta_i^{cd} \check{z}_c(\tau) \check{z}_d(\tau) + \sum_{d=1}^{M} g_i^c (1-g_i^d) \theta_i^{cd} \check{z}_c(\tau) \check{z}_d(\tau) \right) \\
&\quad + \sum_{c=1}^{M} \left(\sum_{d=1}^{M} (1-g_i^c) g_i^d \theta_i^{cd} \check{z}_c(\tau) \check{z}_d(\tau) + \sum_{d=1}^{M} (1-g_i^c)(1-g_i^d) \theta_i^{cd} \check{z}_c(\tau) \check{z}_d(\tau) \right) .
\end{aligned} \tag{S41}$$

Note that

$$\begin{aligned}
g_i^c g_i^d \theta_i^{cd} &= g_i^c g_i^d \\
g_i^c (1-g_i^d) \theta_i^{cd} &= \frac{1}{2} g_i^c (1-g_i^d) \\
(1-g_i^c) g_i^d \theta_i^{cd} &= \frac{1}{2} (1-g_i^c) g_i^d \\
(1-g_i^c)(1-g_i^d) \theta_i^{cd} &= 0
\end{aligned} \tag{S42}$$

where the factor of $\frac{1}{2}$ arises because there is a 50% chance that genotype c (d) with a mutant at locus i and genotype d (c) with a wildtype at locus i will recombine to a genotype with a mutant at locus i. Hence, we can further write (S41) as

$$\begin{aligned}
\sum_{a=1}^{M} g_i^a \sum_{c=1}^{M} \sum_{d=1}^{M} R_{a,cd} \check{z}_c(\tau) \check{z}_d(\tau) &= \sum_{c=1}^{M} \left(\sum_{d=1}^{M} g_i^c g_i^d \check{z}_c(\tau) \check{z}_d(\tau) + \frac{1}{2} \sum_{d=1}^{M} g_i^c (1-g_i^d) \check{z}_c(\tau) \check{z}_d(\tau) \right) \\
&\quad + \frac{1}{2} \sum_{c=1}^{M} \sum_{d=1}^{M} (1-g_i^c) g_i^d \check{z}_c(\tau) \check{z}_d(\tau) \\
&= \check{x}_i^2(\tau) + \frac{1}{2} \check{x}_i(\tau)(1 - x_i(\tau)) + \frac{1}{2} \check{x}_i(\tau)(1 - \check{x}_i(\tau)) \\
&= \check{x}_i(\tau).
\end{aligned} \tag{S43}$$

Substituting (S43) back into (S39), we see that

$$\eta_e(\check{\mathbf{x}}(\tau)) = 0 \qquad \text{for} \quad 1 \le e \le L. \tag{S44}$$

Now consider the case when $L < e \le R$. Developing as in (S39), we get

$$\eta_e(\check{\mathbf{x}}(\tau)) = (L-1)\check{x}_{ij}(\tau) - (L-1)\sum_{a=1}^{M} g_i^a g_j^a \sum_{c=1}^{M}\sum_{d=1}^{M} R_{a,cd}\check{z}_c(\tau)\check{z}_d(\tau). \tag{S45}$$

To simplify, we define $\theta_{ij}^{cd} := \sum_{a=1}^{M} g_i^a g_j^a R_{a,cd}$ where θ_{ij}^{cd} is the probability that genotypes c and d recombine to form a genotype which has a double mutant at locus-pair (i,j). We thus have

$$\sum_{a=1}^{M} g_i^a g_j^a \sum_{c=1}^{M}\sum_{d=1}^{M} R_{a,cd}\check{z}_c(\tau)\check{z}_d(\tau) = \sum_{c=1}^{M}\sum_{d=1}^{M} \theta_{ij}^{cd}\check{z}_c(\tau)\check{z}_d(\tau)\;. \tag{S46}$$

To proceed, it is convenient to first recognize that $R_{a,cd}$, and thus θ_{ij}^{cd}, depend on the number of breakpoints occurring in the recombination event. However, under the small r assumption (S10), it is sufficient to consider only a single breakpoint since the probability of more than one breakpoint is $O(\frac{1}{N^2})$ (see (S12)). By noting that $1 = 1 - g_i^c + g_i^c$, we proceed by dividing the two summations in $\sum_{c=1}^{M}\sum_{d=1}^{M}\theta_{ij}^{cd}\check{z}_c(\tau)\check{z}_d(\tau)$ into 16 summations, corresponding to whether there are mutations at loci i and j in genotypes c and d. Specifically, these 16 summations correspond to the 16 possible allele-pairs in genotypes c and d, shown in the first and second columns of Table S2. We define the 'event' A_{ij}^{cd}, as the event that recombination of genotype c and d results in the locus-pair (i,j) both having mutant alleles. Similar to (S41), we may thus decompose (S46) as

$$\begin{aligned}
\sum_{c=1}^{M}\sum_{d=1}^{M}\theta_{ij}^{cd}\check{z}_c(\tau)\check{z}_d(\tau) = &\sum_c\sum_d \Pr(A_{ij}^{cd})(1-g_i^c)(1-g_j^c)(1-g_i^d)(1-g_j^d)\check{z}_c(\tau)\check{z}_d(\tau)\\
&+\sum_c\sum_d \Pr(A_{ij}^{cd})(1-g_i^c)(1-g_j^c)(1-g_i^d)g_j^d\check{z}_c(\tau)\check{z}_d(\tau)\\
&+\sum_c\sum_d \Pr(A_{ij}^{cd})(1-g_i^c)(1-g_j^c)g_i^d(1-g_j^d)\check{z}_c(\tau)\check{z}_d(\tau)+\sum_c\sum_d \Pr(A_{ij}^{cd})(1-g_i^c)(1-g_j^c)g_i^d g_j^d\check{z}_c(\tau)\check{z}_d(\tau)\\
&+\sum_c\sum_d \Pr(A_{ij}^{cd})(1-g_i^c)g_j^c(1-g_i^d)(1-g_j^d)\check{z}_c(\tau)\check{z}_d(\tau)+\sum_c\sum_d \Pr(A_{ij}^{cd})(1-g_i^c)g_j^c(1-g_i^d)g_j^d\check{z}_c(\tau)\check{z}_d(\tau)\\
&+\sum_c\sum_d \Pr(A_{ij}^{cd})(1-g_i^c)g_j^c g_i^d(1-g_j^d)\check{z}_c(\tau)\check{z}_d(\tau)+\sum_c\sum_d \Pr(A_{ij}^{cd})(1-g_i^c)g_j^c g_i^d g_j^d\check{z}_c(\tau)\check{z}_d(\tau)\\
&+\sum_c\sum_d \Pr(A_{ij}^{cd})g_i^c(1-g_j^c)(1-g_i^d)(1-g_j^d)\check{z}_c(\tau)\check{z}_d(\tau)+\sum_c\sum_d \Pr(A_{ij}^{cd})g_i^c(1-g_j^c)(1-g_i^d)g_j^d\check{z}_c(\tau)\check{z}_d(\tau)\\
&+\sum_c\sum_d \Pr(A_{ij}^{cd})g_i^c(1-g_j^c)g_i^d(1-g_j^d)\check{z}_c(\tau)\check{z}_d(\tau)+\sum_c\sum_d \Pr(A_{ij}^{cd})g_i^c(1-g_j^c)g_i^d g_j^d\check{z}_c(\tau)\check{z}_d(\tau)\\
&+\sum_c\sum_d \Pr(A_{ij}^{cd})g_i^c g_j^c(1-g_i^d)(1-g_j^d)\check{z}_c(\tau)\check{z}_d(\tau)+\sum_c\sum_d \Pr(A_{ij}^{cd})g_i^c g_j^c(1-g_i^d)g_j^d\check{z}_c(\tau)\check{z}_d(\tau)\\
&+\sum_c\sum_d \Pr(A_{ij}^{cd})g_i^c g_j^c g_i^d(1-g_j^d)\check{z}_c(\tau)\check{z}_d(\tau)+\sum_c\sum_d \Pr(A_{ij}^{cd})g_i^c g_j^c g_i^d g_j^d\check{z}_c(\tau)\check{z}_d(\tau).
\end{aligned} \tag{S47}$$

Now using the Total Probability Theorem, we have

$$\begin{aligned}
\Pr\left(A_{ij}^{cd}\right) = &\Pr\left(A_{ij}^{cd}|1<\text{bpt.}<i\right)\times\Pr\left(1<\text{bpt.}<i\right)+\Pr\left(A_{ij}^{cd}|i<\text{bpt.}<j\right)\times\Pr\left(i<\text{bpt.}<j\right)\\
&+\Pr\left(A_{ij}^{cd}|j<\text{bpt.}<L\right)\times\Pr\left(j<\text{bpt.}<L\right),
\end{aligned} \tag{S48}$$

where bpt. stands for breakpoint., $\Pr(i<\text{bpt.}<j)$ is the probability that the breakpoint lies between loci i and j, with $i<j$, and $\Pr\left(A_{ij}^{cd}|i<\text{bpt.}<j\right)$ is the conditional probability that event A_{ij}^{cd} occurs. These probabilities are given in

Table S2 Probabilities of recombination events in (S48). The factor of $\frac{1}{2}$ arises because there is 50% chance of choosing genotypes c or d in the recombination process. The denominator is $L-1$ as there are only $L-1$ possible locations along the sequence length where a breakpoint (bpt.) can occur.

Genotype c locus-pair (i,j)	Genotype d locus-pair (i,j)	$\Pr\left(A_{ij}^{cd}\mid 1<\text{bpt.}<i\right)$ $\times\Pr\left(1<\text{bpt.}<i\right)$	$\Pr\left(A_{ij}^{cd}\mid i<\text{bpt.}<j\right)$ $\times\Pr\left(i<\text{bpt.}<j\right)$	$\Pr\left(A_{ij}^{cd}\mid j<\text{bpt.}<L\right)$ $\times\Pr\left(j<\text{bpt.}<L\right)$
00	00	0	0	0
00	01	0	0	0
00	10	0	0	0
00	11	$\frac{1}{2}\frac{i-1}{L-1}$	0	$\frac{1}{2}\frac{L-j}{L-1}$
01	00	0	0	0
01	01	0	0	0
01	10	0	$\frac{1}{2}\frac{j-i}{L-1}$	0
01	11	$\frac{1}{2}\frac{i-1}{L-1}$	$\frac{1}{2}\frac{j-i}{L-1}$	$\frac{1}{2}\frac{L-j}{L-1}$
10	00	0	0	0
10	01	0	$\frac{1}{2}\frac{j-i}{L-1}$	0
10	10	0	0	0
10	11	$\frac{1}{2}\frac{i-1}{L-1}$	$\frac{1}{2}\frac{j-i}{L-1}$	$\frac{1}{2}\frac{L-j}{L-1}$
11	00	$\frac{1}{2}\frac{i-1}{L-1}$	0	$\frac{1}{2}\frac{L-j}{L-1}$
11	01	$\frac{1}{2}\frac{i-1}{L-1}$	$\frac{1}{2}\frac{j-i}{L-1}$	$\frac{1}{2}\frac{L-j}{L-1}$
11	10	$\frac{1}{2}\frac{i-1}{L-1}$	$\frac{1}{2}\frac{j-i}{L-1}$	$\frac{1}{2}\frac{L-j}{L-1}$
11	11	$\frac{i-1}{L-1}$	$\frac{j-i}{L-1}$	$\frac{L-j}{L-1}$

Table S2, from which we have

$$
\begin{aligned}
\sum_{c=1}^{M}\sum_{d=1}^{M}\theta_{ij}^{cd}\check{z}_c(\tau)\check{z}_d(\tau) = &\sum_{c=1}^{M}\sum_{d=1}^{M}\Pr\left(A_{ij}^{cd}\right)\left(1-g_i^c\right)\left(1-g_j^c\right)g_i^d g_j^d\check{z}_c(\tau)\check{z}_d(\tau)+\sum_{c=1}^{M}\sum_{d=1}^{M}\Pr\left(A_{ij}^{cd}\right)g_i^c g_j^c\left(1-g_i^d\right)\left(1-g_j^d\right)\check{z}_c(\tau)\check{z}_d(\tau)\\
&+\sum_{c=1}^{M}\sum_{d=1}^{M}\Pr\left(A_{ij}^{cd}\right)\left(1-g_i^c\right)g_j^c g_i^d g_j^d\check{z}_c(\tau)\check{z}_d(\tau)+\sum_{c=1}^{M}\sum_{d=1}^{M}\Pr\left(A_{ij}^{cd}\right)g_i^c g_j^c\left(1-g_i^d\right)g_j^d\check{z}_c(\tau)\check{z}_d(\tau)\\
&+\sum_{c=1}^{M}\sum_{d=1}^{M}\Pr\left(A_{ij}^{cd}\right)\left(1-g_i^c\right)g_j^c g_i^d\left(1-g_j^d\right)\check{z}_c(\tau)\check{z}_d(\tau)+\sum_{c=1}^{M}\sum_{d=1}^{M}\Pr\left(A_{ij}^{cd}\right)g_i^c\left(1-g_j^c\right)\left(1-g_i^d\right)g_j^d\check{z}_c(\tau)\check{z}_d(\tau)\\
&+\sum_{c=1}^{M}\sum_{d=1}^{M}\Pr\left(A_{ij}^{cd}\right)g_i^c\left(1-g_j^c\right)g_i^d g_j^d\check{z}_c(\tau)\check{z}_d(\tau)+\sum_{c=1}^{M}\sum_{d=1}^{M}\Pr\left(A_{ij}^{cd}\right)g_i^c g_j^c g_i^d\left(1-g_j^d\right)\check{z}_c(\tau)\check{z}_d(\tau)\\
&+\sum_{c=1}^{M}\sum_{d=1}^{M}\Pr\left(A_{ij}^{cd}\right)g_i^c g_j^c g_i^d g_j^d\check{z}_c(\tau)\check{z}_d(\tau)\\
=\ & 2\times\frac{L-j-i-1}{2(L-1)}\left(\check{x}_{ij}(\tau)-\check{x}_i(\tau)\check{x}_{ij}(\tau)-\check{x}_j(\tau)\check{x}_{ij}(\tau)+\check{x}_{ij}^2(\tau)\right)+2\times\frac{1}{2}\left(\check{x}_j(\tau)\check{x}_{ij}(\tau)-\check{x}_{ij}^2(\tau)\right)\\
&+2\times\frac{j-i}{2(L-1)}\left(\check{x}_i(\tau)\check{x}_j(\tau)-\check{x}_i(\tau)\check{x}_{ij}(\tau)-\check{x}_j(\tau)\check{x}_{ij}(\tau)+\check{x}_{ij}^2(\tau)\right)+2\times\frac{1}{2}\left(\check{x}_i(\tau)\check{x}_{ij}(\tau)-\check{x}_{ij}^2(\tau)\right)+\check{x}_{ij}^2(\tau)\\
=\ & \check{x}_{ij}(\tau)-\frac{j-i}{L-1}\left(\check{x}_{ij}(\tau)-\check{x}_i(\tau)\check{x}_j(\tau)\right).
\end{aligned}
\tag{S49}
$$

Substituting (S49) together with (S46) back into (S45) we see that

$$\eta_e(\check{\mathbf{x}}(\tau)) = (j-i)\big(\check{x}_{ij}(\tau) - \check{x}_i(\tau)\check{x}_j(\tau)\big) \qquad \text{for} \quad L < e \leq R. \tag{S50}$$

Thus, from (S44) and (S50) we have

$$\eta_e(\check{\mathbf{x}}(\tau)) = \begin{cases} 0 & 1 \leq e \leq L \\ (j-i)\big(\check{x}_{ij}(\tau) - \check{x}_i(\tau)\check{x}_j(\tau)\big) & L < e \leq R, \end{cases} \tag{S51}$$

where i and j are the subscript indices corresponding to the eth element of $\check{\mathbf{x}}(\tau)$.

Probability of observing the allele trajectory: Given the drift vector and diffusion matrix, we can directly apply (Risken 1989, eq. 4.109) which gives the transition probability density over Δt generations, valid for small $\delta\tau\Delta t$ (equivalently large $\frac{N}{\Delta t}$), as

$$\phi(\check{\mathbf{x}}(\tau+\delta\tau\Delta t)|\check{\mathbf{x}}(\tau)) \approx \frac{\exp\left(-\frac{1}{4\delta\tau\Delta t}\left(\check{\mathbf{x}}(\tau+\delta\tau\Delta t) - \check{\mathbf{x}}(\tau) - \bar{d}(\check{\mathbf{x}}(\tau))\delta\tau\Delta t\right)^{\mathrm{T}}\bar{C}(\check{\mathbf{x}}(\tau))^{-1}\left(\check{\mathbf{x}}(\tau+\delta\tau\Delta t) - \check{\mathbf{x}}(\tau) - \bar{d}(\check{\mathbf{x}}(\tau))\delta\tau\Delta t\right)\right)}{(4\pi\delta\tau\Delta t)^{R/2}\sqrt{\det(\bar{C}(\check{\mathbf{x}}(\tau)))}}. \tag{S52}$$

Thus, the transition probability for a single generation of the original discrete-time discrete-frequency WF process can (for large $\frac{N}{\Delta t}$) be approximated by

$$P(\mathbf{x}(t_{k+1})|\mathbf{x}(t_k)) \approx \phi(\mathbf{x}(t_{k+1})|\mathbf{x}(t_k)) \prod_{e=1}^{R} \mathrm{d}x_e(t_{k+1}) \tag{S53}$$

where the $\mathrm{d}x_e$ represent small frequency differences accounting for the quantization of the continuous eth marginal allele frequency space at each time point. The probability of observing a trajectory of mutant allele frequencies $(\mathbf{x}(t_1), \mathbf{x}(t_2), \ldots, \mathbf{x}(t_K))$ conditioned on $\mathbf{x}(t_0)$ is then given by

$$\begin{aligned} P\left((\mathbf{x}(t_k))_{k=1}^{K}|\mathbf{x}(t_0)\right) &= \prod_{k=0}^{K-1} P(\mathbf{x}(t_{k+1})|\mathbf{x}(t_k)) \\ &\approx \prod_{k=0}^{K-1}\left[\frac{1}{\sqrt{\det C(\mathbf{x}(t_k))}}\left(\frac{N}{2\pi\Delta t_k}\right)^{R/2}\prod_{e=1}^{R}\mathrm{d}x_e(t_{k+1})\right]\exp\left(-\frac{N}{2}S\left((\mathbf{x}(t_k))_{k=0}^{K}\right)\right) \end{aligned} \tag{S54}$$

where

$$S\left((\mathbf{x}(t_k))_{k=0}^{K}\right) = \sum_{k=0}^{K-1}\frac{1}{\Delta t_k}\sum_{e=1}^{R}\sum_{f=1}^{R}\left[\mathrm{x}_e(t_{k+1}) - \mathrm{x}_e(t_k) - \mathrm{d}_e(\mathbf{x}(t_k))\Delta t_k\right]\left(\mathrm{C}^{-1}(\mathbf{x}(t_k))\right)_{ef}\left[\mathrm{x}_f(t_{k+1}) - \mathrm{x}_f(t_k) - \mathrm{d}_f(\mathbf{x}(t_k))\Delta t_k\right],$$

which is the desired path integral representation. Note we have defined $\mathrm{d}_e(\mathbf{x}(t_k)) := \frac{\bar{\mathrm{d}}_e(\mathbf{x}(t_k))}{N}$ and $(\mathrm{C}(\mathbf{x}(t_k)))_{ef} := 2\left(\bar{\mathrm{C}}(\mathbf{x}(t_k))\right)_{ef}$.

Maximum a posteriori estimate of allele selection coefficients and epistasis terms

The maximum a posteriori (MAP) estimate of the allele selection coefficients and epistasis terms is obtained by solving

$$\hat{\mathbf{s}} = \arg\max_{\mathbf{s}} \mathfrak{L}\left(\mathbf{s}; \mu, r, N, (\mathbf{x}(t_k))_{k=0}^{K}\right) P^{\text{prior}}(\mathbf{s}), \tag{S55}$$

where

$$\mathfrak{L}\left(\mathbf{s}; \mu, r, N, (\mathbf{x}(t_k))_{k=0}^{K}\right) = \left(\prod_{k=0}^{T-1}\frac{1}{\sqrt{\det C(\mathbf{x}(t_k))}}\left(\frac{N}{2\pi\Delta t_k}\right)^{R/2}\prod_{i=1}^{R}\mathrm{d}x_i(t_{k+1})\right)\prod_{k=0}^{K-1}\exp\left(-\frac{N}{2}S\left((\mathbf{x}(t_k))_{k=0}^{K}\right)\right) \tag{S56}$$

is the (approximate) path-likelihood and

$$P^{\text{prior}}(\mathbf{s}) = \frac{1}{(2\pi\sigma^2)^{R/2}}\exp\left(-\frac{1}{2\sigma^2}\mathbf{s}^{\mathrm{T}}\mathbf{s}\right) \tag{S57}$$

is the assumed prior with $\sigma^2 \in \mathbb{R}$. For convenience, we work with the natural logarithm of the above, i.e.,

$$\ln\left(\mathfrak{L}\left(\mathbf{s};\mu,r,N,\left(\mathbf{x}(t_k)\right)_{k=0}^{K}\right)\right)+\ln\left(P^{\text{prior}}(\mathbf{s})\right)=\ln c_1-\frac{N}{2}\sum_{k=0}^{K-1}S\left(\left(\mathbf{x}(t_k)\right)_{k=0}^{K}\right)+\ln c_2-\frac{1}{2\sigma^2}\mathbf{s}^{\mathrm{T}}\mathbf{s}, \tag{S58}$$

where c_1 and c_2 represent terms that are independent of $\mathbf{s}$. Next, we take the vector partial derivative with respect to $\mathbf{s}$ and equate it to zero to find the MAP estimate of $\mathbf{s}$. This gives

$$\begin{aligned}\mathbf{0}&=\frac{\partial}{\partial\mathbf{s}}\ln c_1-\frac{\partial}{\partial\mathbf{s}}\frac{N}{2}\sum_{k=0}^{K-1}S\left(\left(\mathbf{x}(t_k)\right)_{k=0}^{K}\right)+\frac{\partial}{\partial\mathbf{s}}\ln c_2-\frac{\partial}{\partial\mathbf{s}}\frac{1}{2\sigma^2}\mathbf{s}^{\mathrm{T}}\mathbf{s}\\&=\sum_{k=0}^{K-1}C(\mathbf{x}(t_k))\left[C(\mathbf{x}(t_k))\right]^{-1}\left[\mathbf{x}(t_{k+1})-\mathbf{x}(t_k)-\Delta t_k C(\mathbf{x}(t_k))\mathbf{s}-\mu\Delta t_k\mathbf{v}(\mathbf{x}(t_k))-r\Delta t_k\eta(\mathbf{x}(t_k))\right]+\gamma\,\mathbf{s},\end{aligned} \tag{S59}$$

where $\gamma = 1/N\sigma^2$. Solving the above yields the desired MPL estimator (21), i.e.,

$$\hat{\mathbf{s}}=\left[\sum_{k=0}^{K-1}\Delta t_k C(\mathbf{x}(t_k))+\gamma I\right]^{-1}\left[\mathbf{x}(t_K)-\mathbf{x}(t_0)-\mu\sum_{k=0}^{K-1}\Delta t_k\mathbf{v}(\mathbf{x}(t_k))-r\sum_{k=0}^{K-1}\Delta t_k\boldsymbol{\eta}(\mathbf{x}(t_k))\right]. \tag{S60}$$

We note that, in practice, it is not required to know the exact values of N or σ^2. Rather what is important is that their product γ has an appropriate strength, and this can be treated as a regularization parameter.

Multiple replicates

The inference framework may be applied to incorporate observations of mutant allele frequencies from multiple independent replicates. These replicates may be parallel evolutionary experiments or time-series data from distinct studies. Each replicate represents a unique evolutionary path that may have different initial conditions and/or sampling parameters, independent from the other replicates. Here we give the specific generalization for the bi-allelic model with symmetric mutation probabilities, as considered in Materials and Methods. Further extension to multi-allele and asymmetric mutation probability models is straightforward.

For a scenario with Q replicates, the MAP estimate of the selection coefficients is the solution to

$$\hat{\mathbf{s}}=\arg\max_{\mathbf{s}}\mathfrak{L}\left(\mathbf{s};\mu,N,(\mathbf{x}^1(t_k^1))_{k=0}^{K_1},\cdots,(\mathbf{x}^Q(t_k^Q))_{k=0}^{K_Q}\right)P^{\text{prior}}(\mathbf{s}), \tag{S61}$$

where $\mathbf{x}^q(t_k^q)=(x_1^q(t_k^q),\cdots,x_L^q(t_k^q))$ is the observed mutant allele frequencies at generation t_k^q of replicate q. The likelihood function admits

$$\mathfrak{L}\left(\mathbf{s};\mu,N,(\mathbf{x}^1(t_k^1))_{k=0}^{K_1},\cdots,(\mathbf{x}^Q(t_k^Q))_{k=0}^{K_Q}\right)=\prod_{q=1}^{Q}\prod_{k=0}^{K_q-1}P\left(\mathbf{x}^q(t_{k+1}^q)|\mathbf{x}^q(t_k^q),N,\mu,\mathbf{s}\right) \tag{S62}$$

and, as before, the prior is

$$P^{\text{prior}}(\mathbf{s})=\frac{1}{(2\pi\sigma^2)^{L/2}}\exp\left(-\frac{1}{2\sigma^2}\mathbf{s}^{\mathrm{T}}\mathbf{s}\right). \tag{S63}$$

Using (S54), we obtain the path integral approximation to the likelihood function

$$\begin{aligned}&\mathfrak{L}\left(\mathbf{s};\mu,N,(\mathbf{x}^1(t_k^1))_{k=0}^{K_1},\cdots,(\mathbf{x}^Q(t_k^Q))_{k=0}^{K_Q}\right)\\&\approx\prod_{q=1}^{Q}\left(\prod_{k=0}^{K_q-1}\frac{1}{\sqrt{\det C(\mathbf{x}^q(t_k^q))}}\left(\frac{N}{2\pi\Delta t_k^q}\right)^{L/2}\prod_{i=1}^{L}\mathrm{d}x_i^q(t_{k+1}^q)\right)\exp\left(-\frac{N}{2}S\left((\mathbf{x}^q(t_k^q))_{k=0}^{K_q}\right)\right).\end{aligned} \tag{S64}$$

Substituting this approximation in (S61), we get the MPL estimator of (22), i.e.,

$$\hat{\mathbf{s}}=\left[\sum_{q=1}^{Q}\sum_{k=0}^{K_q-1}\Delta t_k^q C(\mathbf{x}^q(t_k^q))+\gamma I\right]^{-1}\times\left(\sum_{q=1}^{Q}\left[\mathbf{x}^q(t_{K_q}^q)-\mathbf{x}^q(t_0^q)-\mu\sum_{k=0}^{K_q-1}\Delta t_k^q\mathbf{v}(\mathbf{x}^q(t_k^q))-r\sum_{k=0}^{K_q-1}\Delta t_k^q\boldsymbol{\eta}(\mathbf{x}^q(t_k^q))\right]\right), \tag{S65}$$

where $C(\mathbf{x}^q(t_k^q))$ is the covariance matrix of the mutant allele frequencies at generation t_k^q of the qth replicate, $\gamma = 1/N\sigma^2$ as before, and $\Delta t_k^q = t_{k+1}^q - t_k^q$. For each replicate q, the entries of the covariance matrix are computed according to (16)-(18).

Asymmetrical mutation probabilities

So far we have assumed the forward and backward mutation probabilities are equal. The MPL framework can easily accommodate asymmetrical mutation probabilities as was also shown in Sohail *et al.* (2021) for the additive fitness model case. Here, we derive the expression of the drift vector of the allele-level diffusion process for a fitness model with pairwise epistasis terms. The diffusion matrix, being independent of the mutation probability, remains unchanged.

We begin by defining $\mu_{01,i}$ and $\mu_{10,i}$ as the mutation probabilities, at locus i, of the WT allele mutating to mutant allele and the mutant allele mutating to the WT allele respectively. Similar to (S10), as $N \to \infty$

$$\mu_{01,i} = \frac{\bar{\mu}_{01,i}}{N} + O\Big(\frac{1}{N^2}\Big), \quad \mu_{10,i} = \frac{\bar{\mu}_{10,i}}{N} + O\Big(\frac{1}{N^2}\Big), \tag{S66}$$

and consequently

$$\mu_{ab} = \frac{\bar{\mu}_{ab}}{N} + O\Big(\frac{1}{N^2}\Big), \tag{S67}$$

where $\bar{\mu}_{01,i}$, $\bar{\mu}_{10,i}$, and $\bar{\mu}_{ab}$ are constants independent of N.

The ath entry of the drift vector $\bar{d}(\check{\mathbf{z}}(\tau))$ characterizing the genotype-level diffusion process in the case of equal forward and backward mutation probabilities was given by (S15). In the scenario with locus specific unequal forward and backward mutation probabilities, the ath entry of the drift vector is given as

$$\bar{d}_a(\check{\mathbf{z}}(\tau)) = \check{z}_a(\tau)\left(\bar{h}_a - \sum_{b=1}^{M}\bar{h}_b\check{z}_b(\tau)\right) + \left(\sum_{b=1,d_{ab}=1}^{M}\bar{\mu}_{ba}\check{z}_b(\tau) - \sum_{b=1,d_{ab}=1}^{M}\bar{\mu}_{ab}\check{z}_a(\tau)\right) - \bar{r}(L-1)\big(\check{z}_a(\tau) - \psi_a\left(\check{\mathbf{z}}(\tau)\right)\big). \tag{S68}$$

Following the same steps as before, the eth element of the allele-level drift vector is defined as

$$\begin{aligned}
\bar{\mathrm{d}}_e(\check{\mathbf{x}}(\tau)) &:= \sum_{a=1}^{M}\mathrm{u}_e^a\bar{d}_a(\check{\mathbf{z}}(\tau)) \\
&= \sum_{a=1}^{M}\mathrm{u}_e^a\left(\check{z}_a(\tau)\left(\bar{h}_a - \sum_{b=1}^{M}\bar{h}_b\check{z}_b(\tau)\right) + \left(\sum_{b=1,d_{ab}=1}^{M}\bar{\mu}_{ba}\check{z}_b(\tau) - \sum_{b=1,d_{ab}=1}^{M}\bar{\mu}_{ab}\check{z}_a(\tau)\right) - \bar{r}(L-1)\big(\check{z}_a(\tau) - \psi_a\left(\check{\mathbf{z}}(\tau)\right)\big)\right) \\
&= \sum_{a=1}^{M}\mathrm{u}_e^a\left(\check{z}_a(\tau)(1-\check{z}_a(\tau))\bar{h}_a - \check{z}_a(\tau)\sum_{b=1,b\neq a}^{M}\bar{h}_b\check{z}_b(\tau) + \left(\sum_{b=1,d_{ab}=1}^{M}\bar{\mu}_{ba}\check{z}_b(\tau) - \sum_{b=1,d_{ab}=1}^{M}\bar{\mu}_{ab}\check{z}_a(\tau)\right) - \bar{r}(L-1)\big(\check{z}_a(\tau) - \psi_a(\check{\mathbf{z}}(\tau)\big)\right) \\
&= \check{\mathrm{x}}_e(\tau)\,(1-\check{\mathrm{x}}_e(\tau))\,\bar{\mathrm{s}}_e + \sum_{f\neq e}\bar{\mathrm{C}}_{ef}(\check{\mathbf{x}}(\tau))\bar{\mathrm{s}}_f + \Omega_e + \bar{r}\,\eta_e(\check{\mathbf{x}}(\tau)),
\end{aligned} \tag{S69}$$

where $\eta_e(\check{\mathbf{x}}(\tau))$ is given by (S51) and Ω_e is the mutation term in the asymmetrical mutation probabilities scenario. As in case of symmetrical mutation probabilites, we first consider the case $1 \leq e \leq L$, for which

$$\begin{aligned}
\Omega_e &= \sum_{a=1}^{M}\mathrm{u}_e^a\left(\sum_{b=1,d_{ab}=1}^{M}\bar{\mu}_{ba}\check{z}_b(\tau) - \sum_{b=1,d_{ab}=1}^{M}\bar{\mu}_{ab}\check{z}_a(\tau)\right) \\
&= \sum_{a=1}^{M}g_i^a\left(\sum_{b=1,d_{ab}=1}^{M}\bar{\mu}_{ba}\check{z}_b(\tau) - \sum_{b=1,d_{ab}=1}^{M}\bar{\mu}_{ab}\check{z}_a(\tau)\right) \\
&= \sum_{a=1}^{M}\left(\sum_{b=1,d_{ab}=1}^{M}\bar{\mu}_{ba}g_i^a(1-g_i^b)\check{z}_b(\tau) + \sum_{b=1,d_{ab}=1}^{M}\bar{\mu}_{ba}g_i^ag_i^b\check{z}_b(\tau) - \sum_{b=1,d_{ab}=1}^{M}\bar{\mu}_{ab}g_i^a(1-g_i^b)\check{z}_a(\tau) - \sum_{b=1,d_{ab}=1}^{M}\bar{\mu}_{ab}g_i^ag_i^b\check{z}_a(\tau)\right) \\
&= \sum_{a=1}^{M}\sum_{b=1,d_{ab}=1}^{M}g_i^a(1-g_i^b)\left(\bar{\mu}_{ba}\check{z}_b(\tau) - \bar{\mu}_{ab}\check{z}_a(\tau)\right) + \sum_{a=1}^{M}\sum_{b=1,d_{ab}=1}^{M}g_i^ag_i^b\left(\bar{\mu}_{ba}\check{z}_b(\tau) - \bar{\mu}_{ab}\check{z}_a(\tau)\right),
\end{aligned} \tag{S70}$$

where the second last line above follows from noting that the first summation on the right side of the third last line can be decomposed into two parts. The first where genotypes a and b differ only at locus i, and hence mutation of genotype b to genotype a changes the mutant allele frequency at locus i. The second is where the two genotypes differ from each other at a locus other than i and hence a mutation from genotype b to a does not effect the mutant allele frequency at locus i. Similarly, the second summation in the second last line can also be split into two parts. Now, note that

$$\sum_{a=1}^{M}\sum_{b=1,d_{ab}=1}^{M}g_i^ag_i^b\left(\bar{\mu}_{ba}\check{z}_b(\tau) - \bar{\mu}_{ab}\check{z}_a(\tau)\right) = 0,$$

as this quantity represents the mutation of those genotypes b to genotype a where both a and b have the mutant allele at locus i, while

$$\sum_{a=1}^{M}\sum_{b=1,d_{ab}=1}^{M} g_i^a(1-g_i^b)\left(\bar{\mu}_{ba}\check{z}_b(\tau)-\bar{\mu}_{ab}\check{z}_a(\tau)\right)=\bar{\mu}_{01,i}\left(1-\check{x}_i(\tau)\right)-\bar{\mu}_{10,i}\check{x}_i(\tau), \tag{S71}$$

which represents the flow of mutational probabilities between the WT and the mutation allele, i.e., the mutational flux. Here $\mu_{\alpha\beta,i}$ is the per generation probability of allele α mutating to allele β at locus i.

Now consider the case when $L < e \leq R$ where we have

$$\begin{aligned}\Omega_e &= \sum_{a=1}^{M} \mathrm{u}_e^a\left(\sum_{b=1,d_{ab}=1}^{M}\bar{\mu}_{ba}\check{z}_b(\tau)-\sum_{b=1,d_{ab}=1}^{M}\bar{\mu}_{ab}\check{z}_a(\tau)\right)\\
&= \sum_{a=1}^{M} g_i^a g_j^a\left(\sum_{b=1,d_{ab}=1}^{M}\bar{\mu}_{ba}\check{z}_b(\tau)-\sum_{b=1,d_{ab}=1}^{M}\bar{\mu}_{ab}\check{z}_a(\tau)\right)\\
&= \sum_{a=1}^{M}\sum_{b=1,d_{ab}=1}^{M} g_i^a g_j^a(1-g_i^b)(1-g_j^b)\left(\bar{\mu}_{ba}\check{z}_b(\tau)-\bar{\mu}_{ab}\check{z}_a(\tau)\right)+\sum_{a=1}^{M}\sum_{b=1,d_{ab}=1}^{M} g_i^a g_j^a(1-g_i^b)g_j^b\left(\bar{\mu}_{ba}\check{z}_b(\tau)-\bar{\mu}_{ab}\check{z}_a(\tau)\right)\\
&\quad+\sum_{a=1}^{M}\sum_{b=1,d_{ab}=1}^{M} g_i^a g_j^a g_i^b(1-g_j^b)\left(\bar{\mu}_{ba}\check{z}_b(\tau)-\bar{\mu}_{ab}\check{z}_a(\tau)\right)+\sum_{a=1}^{M}\sum_{b=1,d_{ab}=1}^{M} g_i^a g_j^a g_i^b g_j^b\left(\bar{\mu}_{ba}\check{z}_b(\tau)-\bar{\mu}_{ab}\check{z}_a(\tau)\right).\end{aligned} \tag{S72}$$

Here, the summations on the right side of the second last line above represent the net mutational flow to genotypes that contain alleles $(1,1)$ at locus-pair (i,j), from those genotypes that have alleles $(0,0)$, $(0,1)$, $(1,0)$ and $(1,1)$ respectively at locus-pair (i,j). We note that

$$\begin{aligned}\sum_{a=1}^{M}\sum_{b=1,d_{ab}=1}^{M} g_i^a g_j^a(1-g_i^b)(1-g_j^b)\left(\bar{\mu}_{ba}\check{z}_b(\tau)-\bar{\mu}_{ab}\check{z}_a(\tau)\right)&=0\\
\sum_{a=1}^{M}\sum_{b=1,d_{ab}=1}^{M} g_i^a g_j^a(1-g_i^b)g_j^b\left(\bar{\mu}_{ba}\check{z}_b(\tau)-\bar{\mu}_{ab}\check{z}_a(\tau)\right)&=\bar{\mu}_{01,i}\check{x}_{ij}^{01}(\tau)-\bar{\mu}_{10,i}\check{x}_{ij}^{11}(\tau)\\
\sum_{a=1}^{M}\sum_{b=1,d_{ab}=1}^{M} g_i^a g_j^a g_i^b(1-g_j^b)\left(\bar{\mu}_{ba}\check{z}_b(\tau)-\bar{\mu}_{ab}\check{z}_a(\tau)\right)&=\bar{\mu}_{10,j}\check{x}_{ij}^{01}(\tau)-\bar{\mu}_{10,j}\check{x}_{ij}^{11}(\tau)\\
\sum_{a=1}^{M}\sum_{b=1,d_{ab}=1}^{M} g_i^a g_j^a g_i^b g_j^b\left(\bar{\mu}_{ba}\check{z}_b(\tau)-\bar{\mu}_{ab}\check{z}_a(\tau)\right)&=0.\end{aligned}$$

Substituting the above in (S72) we have

$$\begin{aligned}\Omega_e &= \bar{\mu}_{01,i}\check{x}_{ij}^{01}(\tau)-\bar{\mu}_{10,i}\check{x}_{ij}^{11}(\tau)+\bar{\mu}_{10,j}\check{x}_{ij}^{01}(\tau)-\bar{\mu}_{10,j}\check{x}_{ij}^{11}(\tau)\\
&= \bar{\mu}_{01,i}\left(\check{x}_j^{1}(\tau)-\check{x}_{ij}^{11}(\tau)\right)-\bar{\mu}_{10,i}\check{x}_{ij}^{11}(\tau)+\bar{\mu}_{01,j}\left(\check{x}_i^{1}(\tau)-\check{x}_{ij}^{11}(\tau)\right)-\bar{\mu}_{10,j}\check{x}_{ij}^{11}(\tau).\end{aligned} \tag{S73}$$

Dropping the superscripts from $\check{x}_i(\tau)$ and $\check{x}_{ij}(\tau)$ as before, and from (S71) and (S73), we have

$$\Omega_e=\bar{\mu}_{01,i}\mathrm{v}_e'(\check{\mathbf{x}}(\tau))-\bar{\mu}_{10,i}\mathrm{v}_e''(\check{\mathbf{x}}(\tau))+\bar{\mu}_{01,j}\mathrm{w}_e'(\check{\mathbf{x}}(\tau))-\bar{\mu}_{10,j}\mathrm{w}_e''(\check{\mathbf{x}}(\tau)) \tag{S74}$$

where

$$\mathrm{v}_e'(\check{\mathbf{x}}(\tau))=\begin{cases}1-\check{x}_i(\tau) & 1\leq e\leq L\\ \check{x}_j(\tau)-\check{x}_{ij}(\tau) & L<e\leq R\end{cases}$$

$$\mathrm{v}_e''(\check{\mathbf{x}}(\tau))=\begin{cases}\check{x}_i(\tau) & 1\leq e\leq L\\ \check{x}_{ij}(\tau) & L<e\leq R\end{cases}$$

$$\mathrm{w}_e'(\check{\mathbf{x}}(\tau))=\begin{cases}0 & 1\leq e\leq L\\ \check{x}_i(\tau)-\check{x}_{ij}(\tau) & L<e\leq R,\end{cases}$$

and

$$\mathrm{w}_e''(\check{\mathbf{x}}(\tau)) = \begin{cases} 0 & 1 \leq e \leq L \\ \check{x}_{ij}(\tau) & L < e \leq R. \end{cases}$$

Here (S74) is the mutational term in the asymmetrical mutation probabilities case.

Following similar steps as before, one can derive the MPL estimate with asymmetrical mutation probabilities as

$$\hat{\mathrm{s}}_e = \sum_{f=1}^{R} \left[\sum_{k=0}^{K-1} \Delta t_k C(\mathbf{x}(t_k)) + \gamma I \right]^{-1}_{ef} \left[\mathrm{x}_f(t_K) - \mathrm{x}_f(t_0) - \mu_{01,i} \sum_{k=0}^{K-1} \Delta t_k \mathrm{v}_f'(\mathbf{x}(t_k)) + \mu_{10,i} \sum_{k=0}^{K-1} \Delta t_k \mathrm{v}_f''(\mathbf{x}(t_k)) \right.$$
$$\left. - \mu_{01,j} \sum_{k=0}^{K-1} \Delta t_k \mathrm{w}_f'(\mathbf{x}(t_k)) + \mu_{10,j} \sum_{k=0}^{K-1} \Delta t_k \mathrm{w}_f''(\mathbf{x}(t_k)) - r \sum_{k=0}^{K-1} \Delta t_k \eta_f(\mathbf{x}(t_k)) \right]. \tag{S75}$$

Robustness

Numerical issues may arise in computing the estimate in (S75) in scenarios with severe data limitations (low number of samples, large time between samples). These can be addressed by assuming the allele frequency trajectories are piecewise continuous and the covariance matrix, $C(\mathbf{x}(t_k))$, is also a piecewise continuous function. This allows to replace the summation over time in (S75) with integration, which can then be computed analytically. Specifically, the diagonal terms of the integrated covariance matrix are

$$\frac{(3 - 2\,\mathrm{x}_e(t_{k+1}))\,(\mathrm{x}_e(t_k) + \mathrm{x}_e(t_{k+1}))}{6} - \frac{\mathrm{x}_e^2(t_k)}{3}, \tag{S76}$$

and the off-diagonal terms of the integrated covariance matrix are

$$\frac{\mathrm{x}_{ef}(t_k) + \mathrm{x}_{ef}(t_{k+1})}{2} - \left(\frac{\mathrm{x}_e(t_k)\mathrm{x}_f(t_k)}{3} + \frac{\mathrm{x}_e(t_{k+1})\mathrm{x}_f(t_{k+1})}{3} \right.$$
$$\left. + \frac{\mathrm{x}_e(t_k)\mathrm{x}_f(t_{k+1})}{6} + \frac{\mathrm{x}_e(t_{k+1})\mathrm{x}_f(t_k)}{6} \right), \tag{S77}$$

where the same mapping holds for indices e and f in (S76) and (S77) as in (16)-(18). The mutation terms are now given as

$$\mathrm{v}_f'(.) = \begin{cases} 1 - \frac{x_i(t_k)+x_i(t_{k+1})}{2} & 1 \leq f \leq L \\ \frac{x_j(t_k)+x_j(t_{k+1})}{2} - \frac{x_{ij}(t_k)+x_{ij}(t_{k+1})}{2} & L < f \leq R \end{cases}$$

$$\mathrm{v}_f''(.) = \begin{cases} \frac{x_i(t_k)+x_i(t_{k+1})}{2} & 1 \leq f \leq L \\ \frac{x_{ij}(t_k)+x_{ij}(t_{k+1})}{2} & L < f \leq R \end{cases}$$

$$\mathrm{w}_f'(.) = \begin{cases} 0 & 1 \leq f \leq L \\ \frac{x_i(t_k)+x_i(t_{k+1})}{2} - \frac{x_{ij}(t_k)+x_{ij}(t_{k+1})}{2} & L < f \leq R, \end{cases}$$

and

$$\mathrm{w}_f''(.) = \begin{cases} 0 & 1 \leq f \leq L \\ \frac{x_{ij}(t_k)+x_{ij}(t_{k+1})}{2} & L < f \leq R. \end{cases}$$

While the recombination term $\eta_f(.)$ is 0 for $1 \leq e \leq L$ and given by

$$(i - j) \left(\frac{x_{ij}(t_k) + x_{ij}(t_{k+1})}{2} - \left(\frac{x_i(t_k)x_j(t_k)}{3} \right. \right.$$
$$\left. \left. + \frac{x_i(t_{k+1})x_j(t_{k+1})}{3} + \frac{x_i(t_k)x_j(t_{k+1})}{6} + \frac{x_i(t_{k+1})x_j(t_k)}{6} \right) \right) \tag{S78}$$

for $L < e \leq R$.

Equivalence of genotype and allele-level analyses

The MAP estimate of the allele selection coefficients and epistasis terms can also be obtained from the genotype path integral (S17) by solving

$$\hat{\mathbf{s}} = \arg\max_{\mathbf{s}} \mathfrak{L}\left(\mathbf{s}; \mu, r, N, (\mathbf{z}(t_k))_{k=0}^{K}\right) P^{\text{prior}}(\mathbf{s}) \,. \tag{S79}$$

The prior probability, with $\sigma^2 \in \mathbb{R}$, is the same as in (S63) and given below for convenience

$$P^{\text{prior}}(\mathbf{s}) = \frac{1}{(2\pi\sigma^2)^{L/2}} \exp\left(-\frac{1}{2\sigma^2}\mathbf{s}^{\mathrm{T}}\mathbf{s}\right) \,.$$

From (S17), the approximate genotype path-likelihood is given by

$$\mathfrak{L}\left(\mathbf{s}; \mu, r, N, (\mathbf{z}(t_k))_{k=0}^{K}\right) = \left(\prod_{k=0}^{T-1} \frac{1}{\sqrt{\det C(\mathbf{z}(t_k))}} \left(\frac{N}{2\pi\Delta t_k}\right)^{R/2} \prod_{a=1}^{M} \mathrm{d}z_a(t_{k+1})\right) \prod_{k=0}^{K-1} \exp\left(-\frac{N}{2} S\left((\mathbf{z}(t_k))_{k=0}^{K}\right)\right) ,$$

with

$$S\left((\mathbf{z}(t_k))_{k=0}^{K}\right) = \sum_{k=0}^{K-1} \frac{1}{\Delta t_k} \sum_{a=1}^{M}\sum_{b=1}^{M} \left[z_a(t_{k+1}) - z_a(t_k) - d_a(\mathbf{z}(t_k))\Delta t_k\right] \left(C^{-1}(\mathbf{z}(t_k))\right)_{ab} \left[z_b(t_{k+1}) - z_b(t_k) - d_b(\mathbf{z}(t_k))\Delta t_k\right] ,$$

where $\Delta t_k = t_{k+1} - t_k$ and

$$d_a(\mathbf{z}(t_k)) = z_a(t_k)\left(h_a - \sum_{b=1}^{M} h_b z_b(t_k)\right) + \mu\left(\sum_{b=1, d_{ab}=1}^{M} z_b(t_k) - \sum_{b=1, d_{ab}=1}^{M} z_a(t_k)\right) - r(L-1)\left(z_a(t_k) - \psi_a\left(\mathbf{z}(t_k)\right)\right),$$

with

$$C_{ab}(\mathbf{z}(t_k)) = \begin{cases} z_a(t_k)(1 - z_a(t_k)) & a = b \\ -z_a(t_k)z_b(t_k) & a \neq b. \end{cases}$$

Maximizing the natural logarithm of (S79) and taking the vector partial derivative gives

$$\mathbf{0} = \frac{\partial}{\partial \mathbf{s}} \ln c_1 - \frac{\partial}{\partial \mathbf{s}} \frac{N}{2} \sum_{k=0}^{K-1} S\left((\mathbf{z}(t_k))_{k=0}^{K}\right) + \frac{\partial}{\partial \mathbf{s}} \ln c_2 - \frac{\partial}{\partial \mathbf{s}} \frac{1}{2\sigma^2} \mathbf{s}^{\mathrm{T}}\mathbf{s} \,. \tag{S80}$$

We note that h_a can be expressed as

$$h_a = \sum_{e=1}^{R} \mathrm{u}_e^a \mathrm{s}_e,$$

and from (S18) and (S22), that the single and double mutant allele frequencies can be expressed

$$\mathrm{x}_e = \sum_{e=1}^{R} u_e^a z_a(t). \tag{S81}$$

Substituting these transformation in (S80) and using the results in (S30), (S33), (S38), and (S51), we obtain

$$\hat{\mathbf{s}} = \left[\sum_{k=0}^{K-1} \Delta t_k C(\mathbf{x}(t_k)) + \gamma I\right]^{-1} \left[\mathbf{x}(t_K) - \mathbf{x}(t_0) - \mu \sum_{k=0}^{K-1} \Delta t_k \mathbf{v}(\mathbf{x}(t_k)) - r \sum_{k=0}^{K-1} \Delta t_k \boldsymbol{\eta}(\mathbf{x}(t_k))\right] , \tag{S82}$$

which is the same estimator as in (S60) obtained from allele-level path integral.

Simulations

Simulations were carried out on a system with Intel Core i7-6700HQ 2.6 GHz processor and 16 GB of RAM.

Numerical values of fitness parameters used in simulations are either given in captions of the figures where feasible, or listed in Supplementary Table S1 (separate excel file).

Implementation details of IM

We implemented the method of Illingworth *et al.* (2014), termed IM, in Matlab R2017b. The performance was tested on the fitness landscape with all non-zero epistasis terms (Figure 4A). For a direct comparison with MPL, we did not force IM to search over fitness models of varying sparsity, instead we inferred a fully connected fitness model by providing IM the identity of loci under selection. This way the performance of IM was not affected by a model mismatch between the support of the true and inferred fitness landscape.

Literature cited

Ewens WJ. 2012. *Mathematical Population Genetics 1: Theoretical Introduction*. Springer Science & Business Media.

He Z, Beaumont M, Yu F. 2017. Effects of the ordering of natural selection and population regulation mechanisms on Wright-Fisher models. G3: Genes, Genomes, Genetics. 7:2095–2106.

Illingworth CJ, Fischer A, Mustonen V. 2014. Identifying selection in the within-host evolution of influenza using viral sequence data. PLoS Computational Biology. 10:e1003755.

Illingworth CJ, Parts L, Schiffels S, Liti G, Mustonen V. 2011. Quantifying selection acting on a complex trait using allele frequency time series data. Molecular Biology and Evolution. 29:1187–1197.

Kimura M. 1964. Diffusion models in population genetics. Journal of Applied Probability. 1:177–232.

Mustonen V, Lässig M. 2010. Fitness flux and ubiquity of adaptive evolution. Proceedings of the National Academy of Sciences. 107:4248–4253.

Risken H. 1989. *The Fokker-Planck Equation: Methods of Solution and Applications*. Springer-Verlag. second edition.

Schraiber JG. 2014. A path integral formulation of the Wright–Fisher process with genic selection. Theoretical Population Biology. 92:30–35.

Sohail MS, Louie RH, McKay MR, Barton JP. 2021. MPL resolves genetic linkage in fitness inference from complex evolutionary histories. Nature Biotechnology. 39:472–479.

Tataru P, Bataillon T, Hobolth A. 2015. Inference under a Wright-Fisher model using an accurate beta approximation. Genetics. 201:1133–1141.

Tataru P, Simonsen M, Bataillon T, Hobolth A. 2017. Statistical inference in the Wright-Fisher model using allele frequency data. Systematic Biology. 66:e30–e46.
